# Comparisons between Caucasian‐validated photo‐numeric scales and Korean‐validated photo‐numeric scales for photo‐ageing. Insights from the Singapore/Malaysia cross‐sectional genetics epidemiology study (SMCGES) cohort

**DOI:** 10.1111/srt.13637

**Published:** 2024-05-23

**Authors:** Jun Yan Ng, Hongyu Zhou, Tianqi Li, Fook Tim Chew

**Affiliations:** ^1^ Department of Biological Sciences Faculty of Science National University of Singapore Singapore Singapore

**Keywords:** ageing, Caucasian, Chinese, Korean, photo‐ageing, photo‐numeric, Singapore/Malaysia Cross‐sectional Genetics Epidemiology Study cohort, skin, SMCGES, validation

## Abstract

**Background:**

Photo‐ageing is a form of skin ageing which affects the entire face. A photo‐aged skin has a diverse variety of wrinkles and dyspigmentation all over the face. Here, we discuss photo‐ageing on the Chinese skin evaluated using a photo‐numeric scale developed and validated on Caucasian skin (i.e., Caucasian scale) and evaluated using a photo‐numeric scale developed and validated on Korean skin (i.e., Korean scale). The Korean scale can be subdivided into two scales that separately address the wrinkling and dyspigmentation constituents of photo‐ageing.

**Aim:**

As there are currently no photo‐ageing scales for Chinese skin, the main objective of this study is to adapt existing photo‐ageing photo‐numeric scales for use on ethnic Chinese skin.

**Method:**

Three trained assessors studied facial photo‐ageing on 1,081 ethnic Chinese young adults from the Singapore/Malaysia Cross‐sectional Genetics Epidemiology Study (SMCGES) cohort.

**Results:**

All assessors are highly internally consistent (Weighted Kappa (κ_w_) values≥0.952). We found that the Caucasian scale and Korean scale give nearly synonymous results for the wrinkling constituent of photo‐ageing (*R* ^2^ = 0.9386). The two scales are strongly concordant (Spearman's Rank Correlation (ρ) value: 0.62 ± 0.06, *p* = 1.31×10^−84^). A weak‐to‐moderate inter‐scalar level of agreement (Cohen's Kappa (κ) values: 0.38 ± 0.05, *p* = 8.87×10^−53^) persists and is statistically significant after accounting for agreements due to chance. When tested on ethnic Chinese skin, both scales detect photo‐ageing consistently (Area under curve [AUC] values: 0.76‐0.84). Additionally, the Korean scale for the dyspigmentation constituent of photo‐ageing is concordant with both the Caucasian scale (*R* ^2^ = 0.7888) and the Korean scale for the wrinkling constituent of photo‐ageing (*R* ^2^ = 0.7734).

**Conclusion:**

Our results show that the Caucasian scale is suitable for capturing photo‐ageing on Chinese skin, especially wrinkle variations. The Korean dyspigmentation scale supplements the Caucasian scale to capture dyspigmentation patterns on Chinese skin that may be absent on Caucasian skin. Currently, photo‐ageing scales for Chinese skin are absent. When developed, these photo‐ageing scales must be properly validated for their ability to capture photo‐ageing of the entire face.

**Abbreviations**
AUC: area under curve
BMI: body mass index
cm: centimetres
EF: Electronic focus
EOS: Electro‐Optical System
HDB (Public housing): Public housing constructed by the Singapore Housing Development Board
IBM SPSS/PC: International Business Machines Corporation Statistical Package for Social Scientists Personal Computer (SPSS/PC)
IS: Image stabilisation
ISAAC: International Study of Asthma and Allergies in Childhood
kg: kilograms
m^2^
: squared metres
ROC: receiver operator characteristic
SD: standard deviation
SGD: Singapore dollars
SMCGES: Singapore/Malaysia Cross‐sectional Genetics Epidemiology Study
USM: Ultrasonic motor

## 1 INTRODUCTION

Photo‐ageing is a form of skin ageing characterised by wrinkles and dyspigmentation. While wrinkles and dyspigmentation can take place anywhere on the skin, most studies concentrate their efforts on facial skin.

In our recent systematic review of skin ageing phenotypes,^1^ we discussed photo‐ageing in detail and presented an overview of wrinkling diversity and dyspigmentation patterns identified from published literature and reputable medical books.

It has been known for some time that differences in skin ageing progression exists among people. In our recently published meta‐analysis of the risk factors of skin ageing,^2^ we analysed the literature in detail and found that ethnicity plays a significant role in skin ageing progression. Ethnicity‐specific reasons explain why Caucasian skin wrinkles more easily^3^ while Chinese skin becomes dyspigmented more easily with age.^4^

Differences in skin ageing progression across ethnicities explain the emergence of ethnicity‐specific photo‐numeric scales for evaluating photo‐ageing. Photo‐ageing scales are currently available for Caucasian skin and Korean skin but photo‐ageing scales for Chinese skin have not yet been developed. The main objective of this study is to adapt existing photo‐ageing photo‐numeric scales for use on ethnic Chinese skin.

## 2 METHODS

### 2.1 Participant recruitment

This study comprised of participants recruited from the Singapore/Malaysia Cross‐sectional Genetics Epidemiology Study (SMCGES) cohort. We have previously described this cohort in detail elsewhere.^5^, ^6^, ^7^ Briefly, the SMCGES cohort consists of participants from Singapore and Malaysia and has been previously studied in epidemiological and genetic studies of allergic diseases.

Participant recruitment for the previous study (i.e., the epidemiological and genetic study) was conducted through emails and posters from the National University of Singapore, Singapore (2005 to 2023), Universiti Tunku Abdul Rahman (UTAR) Campus, Malaysia (2016 to 2018), and Sunway University, Malaysia (2019 and 2022). These participants were voluntarily recruited through walk‐in.

Participants from collection drives held from 2011 to 2023 who consented to be re‐contacted were invited to participate in this present study (i.e., the skin ageing study). A total of 10,248 participants were invited to participate in the skin ageing study, of which 3,365 completed the study. All 3,365 participants constitute our study population. The demographics of the respondents and non‐respondents are similar (Table 1). From this study population, we randomly selected a subset of 1,081 ethnic Chinese young adult participants (Table 2).

**TABLE 1:** Summary table for demographics drawn from a population of young Singapore and Malaysia adults recruited from the Singapore/Malaysia Cross‐sectional Genetics Epidemiology Study (SMCGES) cohort.

Demographic factor	Subset evaluated	Response	No response
Participants	1081 (100%)	3365 (100%)	6883 (100%)
Mean age (years) ± SD	26.15 ± 8.11	26.30 ± 6.92	27.47 ± 8.10
Mean height (cm) ± SD	166.03 ± 8.60	165.37 ± 8.69	165.99 ± 8.63
Mean weight (kg) ± SD	60.96 ± 12.60	60.86 ± 13.27	61.00 ± 12.37
BMI (kg/m^2^) ± SD	22.02 ± 3.99	22.19 ± 4.93	22.04 ± 3.69
Gender			
Male	420 (38.85%)	1227 (36.46%)	2862 (41.58%)
Female	661 (61.15%)	2138 (63.54%)	4021 (58.42%)
Ethnicity			
Chinese	1081 (100%)	2885 (85.74%)	5727 (83.20%)
Total monthly family income per capita			
Low	117 (10.82%)	386 (11.47%)	1460 (21.21%)
Moderate	275 (25.44%)	774 (23.00%)	2083 (30.26%)
High	226 (20.91%)	792 (23.54%)	1430 (20.78%)
Very high	446 (41.26%)	1396 (41.49%)	1669 (24.25%)
Missing/Invalid	17 (1.57%)	17 (0.51%)	241 (3.50%)
Housing			
Public housing	664 (61.42%)	1794 (53.31%)	3630 (52.74%)
Condominium/private apartment	320 (29.60%)	852 (25.32%)	1545 (22.45%)
Landed property	76 (7.03%)	698 (20.74%)	1415 (20.56%)
Missing/invalid	21 (1.94%)	21 (0.62%)	293 (4.26%)

*Note*: The values after ± are standard deviation values. Missing/Invalid refers to responses that are either left blank or otherwise invalid. Abbreviations: BMI, body mass index; cm, centimetres; m^2^, squared metres; kg, kilograms; SD, Standard deviation.

**TABLE 2:** Summary table for demographics^*^ drawn from a population of young Singapore ethnic Chinese adults recruited from the Singapore/Malaysia Cross‐sectional Genetics Epidemiology Study (SMCGES) cohort.

Demographic factor	Summary
Participants	1081 (100%)
Mean age (years) ± SD	26.15 ± 8.11
Mean height (cm) ± SD	166.03 ± 8.60
Mean weight (kg) ± SD	60.96 ± 12.60
Age range (years)	18 to 73
BMI (kg/m^2^) ± SD	22.02 ± 3.99
Gender	
Male	420 (38.85%)
Female	661 (61.15%)
Ethnicity	
Chinese	1081 (100%)
Total monthly family income per capita	
<SGD2,000	117 (10.82%)
SGD2,000 to <SGD4,000	275 (25.44%)
SGD 4,000 to <SGD6,000	226 (20.91%)
≥SGD 6,000	446 (41.26%)
Missing/Invalid	17 (1.57%)
Housing	
HDB (public housing)	664 (61.42%)
Condominium/private apartment	320 (29.60%)
Landed property	76 (7.03%)
Missing/invalid	21 (1.94%)

* The values after ± are standard deviation values. Missing/Invalid refers to responses that are either left blank or otherwise invalid.

Abbreviations: BMI, body mass index; cm, centimetres; HDB (Public housing), Public housing constructed by the Singapore Housing Development Board; kg, kilograms; m^2^, squared metres; SD, Standard deviation; SGD, Singapore dollars.

The study was conducted in accordance with the Declaration of Helsinki and Good Clinical Practices.

### 2.2 Survey data collection

The SMCGES cohort participants have previously completed a set of investigator‐administered, validated International Study of Asthma and Allergies in Childhood (ISAAC) questionnaires which collated data on their sociodemographic, personal lifestyles, and familial and personal medical history. After completing the ISAAC questionnaires, the same participants signed an informed consent form to participate in this present study (i.e., the skin ageing study) which contains an investigator‐administered skin ageing questionnaire with more personal lifestyle questions (e.g., Fitzpatrick Skin Type).

Participants were asked a set of three questions to assess their Fitzpatrick Skin Type. This set of questions follows the Fitzpatrick Skin Type characterisation guidelines^8^ to a very high degree. Participants were asked the following three questions. The first question is: ‘Which colour describes your skin?’. The collected data falls in one of three categories: white, brown, and black.

The second question adapted the guideline set by Fitzpatrick, 1988 to suit the local context. To find a suitable approximation for ‘about 45 to 60 minutes of noon exposure in northern (20° to 45°) latitudes in the early summer’, we consulted the typical Ultraviolet Radiation and Ultraviolet Index collected by the Singapore National Environmental Agency.^9^ We found that the Ultraviolet Index is graded as ‘Extreme’ from 1pm to 3pm on a typical day. Participants were, therefore, asked the following question: ‘Recall the times when you had 45–60 minutes of exposure to the Sun in Singapore/Malaysia around 1–3pm. Which statement best describes your skin?’. The collected data falls in one of four categories: (1) I will have a painful burn at 24 h and no tan at seven days. (2) A painful burn at 24 h and a light tan at seven days. (3) A slightly tender burn at 24 h and a moderate tan at seven days. (4) No burn at 24 h and a good tan at seven days.

The third question asked was ‘Which statement best describes your skin?’. The collected data falls in one of four categories: (1) I never go out in the direct sunlight, and when I did go out in my youth, I would only burn and peel. I have actually had severe blistering sunburns requiring bed rest for a couple of days. I never tan at all. (2) I am a sunburner and will only develop a light tan after several exposures. (3) I will develop some nontender sunburn after 45 minutes of initial Sun exposure but can develop a quite dark tan. (4) The Sun is not a problem for me; I would burn if I stayed out several hours on the first day, but I never burn if I am out for an hour or less, even on my first exposure. I tan very well.

Using the classification method detailed by Fitzpatrick, 1988, participants were assessed to have one of six possible skin types: Fitzpatrick Skin Type I, II, III, IV, V, or VI.

### 2.3 Image data collection

Investigators acquire photographic documentation of all participants. Photographs are taken using the same camera (Canon EOS 6DII Body with a EF85 f/1.4L IS USM lens) and tripod positioned one metre away from the participant. Photographs are taken to the standards of other recent and similar studies.^10^, ^11^ In brief, photographs are taken with identical camera settings, lighting, and positioning at five angles—*en face*, 45° oblique, and 90° side profiles. A total of five photos are collected from each participant. All evaluations were performed in an indoor environment with standard lighting.

### 2.4 Evaluating photo‐ageing of the skin

Photo‐ageing is evaluated by three trained assessors on multiple validated photo‐numeric scales reported in Table S1.

Before phenotyping the bulk of the participants, 30 participants are randomly selected and their photographs are openly discussed to reach a consensus among all three assessors. This is done to calibrate the assessment scores. After calibration, each of the three trained assessors grades the rest of the participants independently from the other two assessors (Tables S3 and S4). In summary, with the exception of a randomly selected handful of participants (*n* = 30) whose photographs are used for calibration purposes; all the 1,081 participants are assessed three times independently.

### 2.5 Statistical analysis

All the photo‐numeric scales are standardised.

Two‐tailed bivariate correlations for Spearman's Rank correlation (ρ), Cohen's Kappa (κ), and weighted kappa (κ_w_) are calculated using Version 25 of the IBM Statistical Package for Social Scientists (SPSS/PC). Mean values are reported in Tables 3 and S2 while raw values split by assessor are reported in Table S6. Weighted kappa coefficients (κ_w_) for intra‐assessor agreement are reported in Table S5. The strengths are interpreted as follows – 0.00‐0.19: very weak, 0.20‐0.39: weak, 0.40‐0.59: moderate, 0.60‐0.79: strong, and 0.80‐1.00: very strong.

**FIGURE 1:**
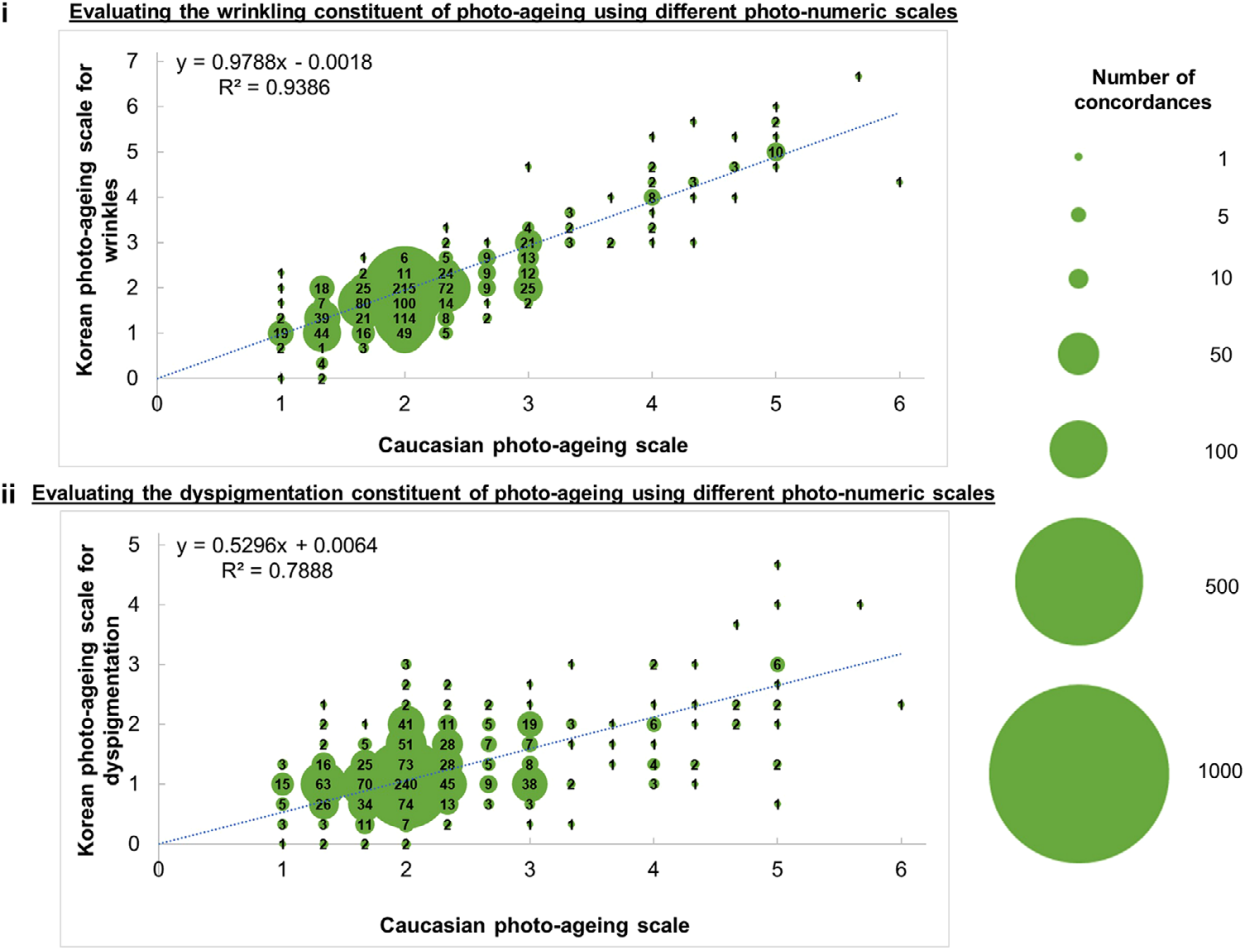
(i) Bubble plots compare the Caucasian photo‐ageing scale and the Korean photo‐ageing scales for wrinkles. Means are calculated from three assessors. Larger circles indicate greater concordance between the two scales. Numbers in the circles are the number of concordances. The sample size of each plot is 1,081 participants. (ii) Bubble plots compare the Caucasian photo‐ageing scale and the Korean photo‐ageing scales for dyspigmentation. Means are calculated from three assessors. Larger circles indicate greater concordance between the two scales. Numbers in the circles are the number of concordances. The sample size of each plot is 1,081 participants.

Bubble plots (Figures 1, S1 and S2) compare the mean scores for photo‐ageing as graded by three independent assessors on four photo‐numeric scales: (i) a Caucasian scale (Griffiths scale), (ii) another Caucasian scale (Larnier scale), a Korean scale that specialises in the wrinkling constituent of photo‐ageing, and (iv) another Korean scale that specialises in the dyspigmentation constituent of photo‐ageing. A linear relationship is assumed for computing the equation for the goodness of fit and the corresponding coefficient of determination (R^2^) value. The strength of the R^2^ value is interpreted as follows – 0.00‐0.19: very weak, 0.20‐0.39: weak, 0.40‐0.59: moderate, 0.60‐0.79: strong, and 0.80‐1.00: very strong.

As there are different Korean photo‐ageing scales for both sexes, we used box and whisker plots split by sex to illustrate the mean severity of photo‐ageing of each sex separately in Figure S3. Box and whisker plots are plotted on Version 4.3.1 of R with the RStudio interface, using the ggplot2 package. Student's T‐Test calculations assume a two‐tailed distribution that is unpaired and has unequal variance (Figure S3).

The area under curve (AUC) of the Receiver Operator Characteristic (ROC) curve is computed using SPSS. Mean values are reported in Tables 3 and S2 while raw values split by assessor are reported in Figures S4 and S5. The strength of the AUC is interpreted as follows – 0.51‐0.59: very weak, 0.60‐0.69: weak, 0.70‐0.79: fair, 0.80‐0.89: strong, and 0.90‐1.00: very strong.

Pair‐wise comparisons were conducted using chi‐square tests to evaluate the proportion of participants with photo‐ageing of different severity levels when stratified by age. The Chi‐square test *p*‐values were corrected for multiple testing using the Bonferroni Correction method. Four photo‐ageing scales were evaluated through this way: (i) the Griffiths scale (Figure S6i), the Larnier scale (Figure S6ii), a Korean scale that specialises in the wrinkling constituent of photo‐ageing (Figure S6iii), and a Korean scale that specialises in the dyspigmentation constituent of photo‐ageing (Figure S6iv). Chi‐square trend tests are performed to evaluate whether the changes in these proportions follow a significant trend with chronological age (Figures S6i to S6iv).

Pair‐wise comparisons were also conducted using chi‐square tests to evaluate the proportion of participants with different Fitzpatrick Skin Type for each photo grade in the Griffiths scale (Figure S7i), the Larnier scale (Figure S7ii), a Korean scale that specialises in the wrinkling constituent of photo‐ageing (Figure S7iii), and a Korean scale that specialises in the dyspigmentation constituent of photo‐ageing (Figure S7iv). The chi‐square test p‐values were corrected for multiple testing using the Bonferroni Correction method.

## 3 RESULTS

### 3.1 Participant demographics

**FIGURE 2:**
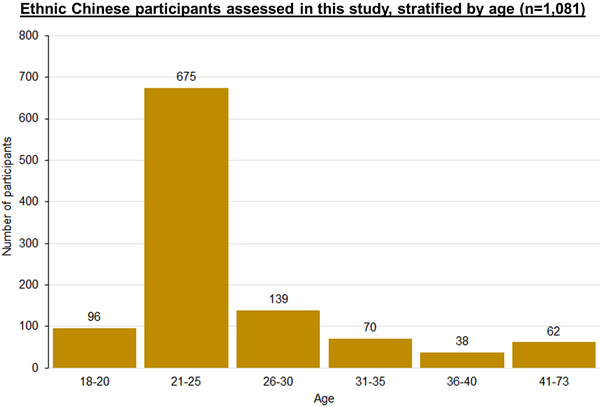
Age distribution of 1,081 ethnic Chinese participants from the Singapore/Malaysia Cross‐sectional Genetics Epidemiology Study (SMCGES) cohort.

Using a sample size of 1,081 participants, we present a representative overview of the epidemiology of skin ageing in the Singapore ethnic Chinese young adult population. Our participants are 166.03 ± 8.60 cm in height, 60.96 ± 12.60 kg in weight, and have a BMI of 22.02 ± 3.99 kg/m^2^. Most participants have a total monthly family income per capita of ≥ 6,000 Singapore dollars (SGD) (*n* = 446, 41.26%). Most participants stay in HDB public housing (*n* = 664, 61.42%) (Table 2). There are more females (*n* = 661, 61.15%) than males in our current study. On average, participants are 26.15 ± 8.11 years old. Our study population consists predominantly of young adults aged 21 to 30. The youngest participant is 18 years’ old, and the oldest participant is 73 years’ old (Figure 2).

Despite being a relatively young study cohort, we report that significant differences exist in the manifestation of photo‐ageing within these young age groups (Figures S6i to S6iv). We treat participants aged 18 to 20 years old as the reference group. When measured on the Griffiths scale, participants aged 21 to 25 years old (*p* = 1.01×10^−6^), participants aged 26 to 30 years old (*p* = 9.33×10^−5^), participants aged 31 to 35 years old (*p* = 5.21×10^−7^), participants aged 36 to 40 years old (*p* = 9.67×10^−11^), and participants aged above 40 years old (*p* = 4.67×10^−108^) exhibit significantly more photo‐ageing as compared to the reference group (Figure S6i). Griffiths scale severity also increases progressively with age (Chi‐square trend test *p* < 0.001) (Figure S6i).

Similar observations are made when photo‐ageing is measured on the Larnier scale. Participants aged 21 to 25 years old (*p* = 1.56×10^−5^), participants aged 26 to 30 years old (*p* = 1.31×10^−5^), participants aged 31 to 35 years old (*p* = 1.57×10^−3^), participants aged 36 to 40 years old (*p* = 6.04×10^−9^), and participants aged above 40 years old (*p* = 1.30×10^−100^) exhibit significantly more photo‐ageing as compared to the reference group (Figure S6ii). Larnier scale severity also increases progressively with age (Chi‐square trend test *p* < 0.001) (Figure S6ii).

When the Korean photo‐ageing scale that specialises in the wrinkling constituent of photo‐ageing was used, similar patterns emerged. Participants aged 21 to 25 years old (*p* = 9.20×10^−3^), participants aged 26 to 30 years old (*p* = 3.98×10^−2^), participants aged 31 to 35 years old (*p* = 5.13×10^−2^), participants aged 36 to 40 years old (*p* = 5.81×10^−10^), and participants aged above 40 years old (*p* = 6.91×10^−73^) exhibit significantly more photo‐ageing as compared to the reference group (Figure S6iii). Photo‐ageing also steadily increases with age (Chi‐square trend test *p* < 0.001) (Figure S6iii).

Lastly, the results using the Korean photo‐ageing scale that specialises in the dyspigmentation constituent of photo‐ageing was similar in this aspect. Participants aged 21 to 25 years old (*p* = 1.01×10^−3^), participants aged 26 to 30 years old (*p* = 1.20×10^−3^), participants aged 31 to 35 years old (*p* = 6.57×10^−5^), participants aged 36 to 40 years old (*p* = 5.02×10^−9^), and participants aged above 40 years old (*p* = 7.63×10^−44^) exhibit significantly more photo‐ageing as compared to the reference group (Figure S6iv). Photo‐ageing also steadily increases with age (Chi‐square trend test *p* < 0.001) (Figure S6iv).

### 3.2 Comparisons between the Caucasian scales and Korean scales

#### 3.2.1 Concordance between Caucasian scales

There are two Caucasian scales for photo‐ageing: the Griffiths scale and the Larnier scale. Both Caucasian scales behave similarly (*R* ^2^ = 0.9352) (Figure S2i, Tables S2 and S6). We selected the Larnier scale rather than the Griffiths scale for further study because the Larnier scale has a simpler relationship with the Korean scale. In detail, an increase of one grade on the Larnier scale is approximately equivalent to an increase of 0.9788 grades on the Korean scale that specialises in the wrinkling constituent of photo‐ageing (Figure 1i). In contrast, an increase of one grade on the Griffiths scale is only approximately equivalent to an increase of 0.8644 grades on the same Korean scale (Figure S2iii). The Larnier scale, not the Griffiths scale, also forms a simpler relationship with the Korean scale that specialises in the dyspigmentation constituent of photo‐ageing (Figure S2iv).

#### 3.2.2 Concordance between Korean scales

There are two Korean scales for photo‐ageing: an unnamed scale that specialises in the wrinkling constituent of photo‐ageing and another unnamed scale that specialises in the dyspigmentation constituent of photo‐ageing. Each scale is further subdivided into sex‐specific scales but these will not be analysed in detail because both sexes behave similarly when assessed on their respective scales (Figure S1). On average, males have more severe skin dyspigmentation patterns than females (Student's T‐Test *p* = 2.40×10^−4^). (Figure S3) but no significant trend is observed for wrinkle variations (Student's T‐Test *p* = 0.376) (Figure S3).

Notable differences exist when photo‐ageing grades on both Korean scales are compared with each other (*R* ^2^ = 0.7734) (Figure S2ii). This shows that both Korean scales excel in achieving their respective design objectives of grading either wrinkle variations or dyspigmentation patterns. Since both Korean scales provide different kinds of phenotypic information, both scales will be analysed in detail.

#### 3.2.3 Level of agreement among assessors

Two kinds of agreement among assessors can be evaluated: intra‐assessor consistency and inter‐assessor consistency. First, we found that each assessor is internally consistent: weighted kappa coefficients (κ_w_) values are very strong (≥0.952) (Table S5). Secondly, we also found that when photo‐ageing is evaluated using the same scale (e.g., the Larnier scale), different assessors grade similarly (Tables S3 and S4).

#### 3.2.4 Concordance and level of agreement between the Caucasian scales and Korean scales

Here, we analyse the suitability of using either the Caucasian or Korean scales for photo‐ageing to evaluate photo‐ageing on the ethnic Chinese face. Our analysis uses the mean assessment grade derived from three independent assessors.

Our results show that the Caucasian scale and the Korean scale are strongly concordant in evaluating the wrinkling constituent of photo‐ageing on the ethnic Chinese facial skin (Spearman ρ = 0.62 ± 0.06, *p* = 1.31×10^−84^) (Table 3). A weak‐to‐moderate level of agreement between both scales persists and is statistically significant after accounting for agreements due to chance (Cohen's κ = 0.38 ± 0.05, *p* = 8.87×10^−53^) (Table 3).

**TABLE 3:** Comparison^*^ between the Caucasian photo‐numeric scale and the Korean photo‐numeric scales for assessing photo‐ageing.

		Comparison	
Measurement		Caucasian photo‐ageing scale and Korean photo‐numeric scale for the wrinkling constituent of photo‐ageing	Caucasian photo‐ageing scale and Korean photo‐numeric scale for the dyspigmentation constituent of photo‐ageing
Mean Spearman's Rank Correlation (ρ)	Value	0.62 ± 0.06	0.32 ± 0.02
	p‐Value	1.31×10^−84^	1.41×10^−23^
Mean Cohen's Kappa (κ)	Value	0.38 ± 0.05	0.11 ± 0.03
	p‐Value	8.87×10^−53^	1.37×10^−5^
Mean area under curve (AUC) of the Receiver Operator Characteristic (ROC) curve	When the Caucasian photo‐numeric scale is the gold standard	0.76 ± 0.05	0.60 ± 0.02
	When the Korean photo‐numeric scale is the gold standard	0.84 ± 0.03	0.64 ± 0.02
Mean Coefficient of determination (*R* ^2^) for the equation for the goodness of fit		0.9386	0.7888
Equation for the goodness of fit		Korean scale = 0.9788 × Caucasian scale ‐ 0.0018	Korean scale = 0.5296 × Caucasian scale + 0.0064

* Means are calculated from three assessors.

We also found a concordance between the Caucasian scale and the Korean scale that specialises in the dyspigmentation constituent of photo‐ageing, though this concordance is weak (Spearman ρ = 0.32 ± 0.02, *p* = 1.41×10^−23^) (Table 3). After adjusting for chanced agreements, a very weak, but statistically significant level of agreement persists between both scales (Cohen's κ = 0.11 ± 0.03, *p* = 1.37×10^−5^) (Table 3).

#### 3.2.5 Goodness of fit between the Caucasian scales and Korean scales

We determined that the Caucasian scale and Korean scale give nearly synonymous results in evaluating the wrinkling constituent of photo‐ageing (*R* ^2^ = 0.9386). The relationship nearly follows a 1:1 ratio: an increase of one grade on the Caucasian scale approximately corresponds to an increase of 0.9788 grades on the Korean scale (Figure 1i). Our results indicate that most participants graded as Grade 1 on the Caucasian scale are also graded as Grade 1 on the Korean scale. The very strong (0.9386) coefficient of determination (R^2^) for the equation for the goodness of fit indicates that the relationship between the Caucasian scale and Korean scale can be very well explained by a linear relationship model.

Next, to evaluate the dyspigmentation constituent of photo‐ageing on Chinese skin, we found that the relationship between the Caucasian scale and Korean scale roughly follows a 2:1 ratio: an increase of two successive grades on the Caucasian scale approximately corresponds to an increase of 1.0592 grades on the Korean scale (*R* ^2^ = 0.7888) (Figure 1ii). Our results show that dyspigmentation patterns are more frequent, more varied, and appear sooner on a given grade of the Korean scale (e.g., Grade 1) when compared to the same grade (i.e., Grade 1) on the Caucasian scale. Thus, the Korean scale enables a stricter characterisation of dyspigmentation progression on facial skin.

#### 3.2.6 Area under curve (AUC) between the Caucasian scales and Korean scales

We first compare how the wrinkling constituent of photo‐ageing is evaluated by the Caucasian scale and the Korean scale. Taking the Caucasian scale as the gold standard, the Korean scale is fairly capable in discerning the severity of photo‐ageing (AUC = 0.76 ± 0.05) (Table 3, Figure S4). In comparison, when the Korean scale is taken as the gold standard, different grades of photo‐ageing can also be strongly distinguishable apart from one another (AUC = 0.84 ± 0.03) (Table 3, Figure S5). This evidence suggests that both the Caucasian scale and the Korean scale are suitable assessment tools to evaluate photo‐ageing on the ethnic Chinese facial skin.

Next, we analyse how the Caucasian and Korean scales compare with each other when we evaluate photo‐ageing based on dyspigmentation patterns. When the Caucasian scale is taken as the gold standard, the Korean scale can weakly discern different grades of photo‐ageing (AUC = 0.60 ± 0.02) (Table 3, Figure S4). Similarly, when we take the Korean scale to be the gold standard, the Caucasian scale is also able to weakly distinguish apart different grades of photo‐ageing (AUC = 0.64 ± 0.02) (Table 3, Figure S5). This evidence suggests that the Korean scale that specialises in the dyspigmentation constituent of photo‐ageing provides sufficiently distinct data that cannot be replicated by the Caucasian scale. This unique data has the potential to add more depth to the multi‐faceted nature of photo‐ageing.

## 4 DISCUSSION

### 4.1 Participant demographics

Singapore is a racially diverse country. This diversity enables us to recruit participants of diverse ethnicities. However, an unequal proportion and sampling probability exists among some races, with only ethnic Chinese remaining as the major racial group with a large enough sample size. While we focused only on the ethnic Chinese here, future analyses and description of skin ageing in other races (e.g., Malays and Indians) will be conducted when the number of participants of the other races grows sufficiently large through progressive, annual recruitment drives to expand the Singapore/Malaysia Cross‐sectional Genetics Epidemiology Study (SMCGES) cohort.

### 4.2 Photo‐ageing scales

Photo‐ageing is a complex phenotype. A photo‐aged skin is a skin characterised by having many types of wrinkles and dyspigmentation all over the face. It has been known for some time in the literature that Caucasian skin wrinkles more easily^3^ while Chinese skin becomes dyspigmented more easily with age.^4^ There are currently no photo‐ageing scales for Chinese skin. The main objective of this study is to adapt existing photo‐ageing photo‐numeric scales for use on ethnic Chinese skin.

We focus our efforts on two available photo‐ageing scales: a widely used scale developed and validated on Caucasian skin (Larnier scale)^12^ and an unnamed scale developed and validated on Korean skin (Jin^13^). The Korean scale is subdivided into two scales, one scale specialises in the wrinkling constituent of photo‐ageing and the other scale specialises in the dyspigmentation constituent of photo‐ageing.

Here, we show a nearly 1:1 match in the grades on the Caucasian scale and the grades on the Korean scale that specialises in the wrinkling constituent of photo‐ageing (approximately 1:0.9788 (*R* ^2^ = 0.9386) (Table 3, Figure 1i)). However, there are noticeable differences between the grades on the Caucasian scale and the grades on the Korean scale that specialises in the dyspigmentation constituent of photo‐ageing (Table 3, Figure 1ii).

We interpret that our results are indicative that the wrinkling constituent of photo‐ageing on Chinese skin can be evaluated equally well on the Caucasian scale and the Korean scale. Since the Caucasian skin is known in the literature to wrinkle more than the skin of other ethnicities, the design of the Caucasian photo‐ageing scale tends to emphasise capturing wrinkles on the face. We have shown that this widely used Caucasian photo‐ageing scale (Larnier scale) is suitable for evaluating photo‐ageing on Chinese skin. Taking into consideration its design objectives, the Caucasian scale may fare better than the Korean scale in capturing the wrinkling constituent of photo‐ageing on Chinese skin.

Since the Chinese skin is known in the literature to become dyspigmented with age more than the skin of other ethnicities,^4^ the Caucasian scale may not sufficiently capture all these variations in dyspigmentation. The Korean scale that specialises in the dyspigmentation aspect of photo‐ageing addresses this shortcoming. The correlation of determination (R^2^) for the equation of goodness of fit between the Larnier scale and the Korean scale that specialises in the dyspigmentation aspect of photo‐ageing (*R* ^2^ = 0.7888) (Figure 1ii) showcases the ability of the Korean scale to identify possibly‐Asian‐specific dyspigmentation patterns over a wide chronological range. Similarly, the R^2^ value between the two Korean scales that specialise in the wrinkling and dyspigmentation constituents of photo‐ageing (*R* ^2^ = 0.7734) (Figure S2ii) shows that the dyspigmentation scale is a sufficiently distinct scale which can function independently.

Working towards achieving our main study objective of adapting existing photo‐ageing photo‐numeric scales for use on ethnic Chinese skin, our results show that the widely used Caucasian scale (Larnier scale) provides ample room to capture wrinkle variations in Chinese skin that may be absent on Korean skin. The Korean dyspigmentation scale can also be independently deployed to supplement the Caucasian scale to capture dyspigmentation in Chinese skin that may be absent on Caucasian skin.

### 4.3 The complexity of a standardised scale for skin photo‐ageing

It is known that a darker skin colour confers greater protection from ultraviolet exposure from the Sun.^14^ Participants with darker skin colour are better protected from photo‐ageing because their skin type is less sensitive to the Sun. The Fitzpatrick Skin Type is a classification system in which participants with Fitzpatrick Skin Types III, IV, V, or VI are better protected from ultraviolet radiation from the Sun and thus, photo‐ageing, as compared to participants with Fitzpatrick Skin Types I or II.^8^

We wondered whether the four photo‐ageing scales studied in this paper considered the effects of skin type when designing their respective photo‐numeric scales. Using the Larnier scale as an example, the proportion of participants with grade 1 photo‐ageing on the Larnier scale and Fitzpatrick Skin Types I through VI are treated as the reference group. Participants with grade 2 photo‐ageing on the Larnier scale have a significantly different composition of Fitzpatrick Skin Types as compared to the reference group (Chi‐square *p* = 3.59×10^−7^). Similar comparisons were made between the reference group and participants with grade 3 photo‐ageing (Chi‐square *p* = 5.68×10^−11^), grade 4 photo‐ageing (Chi‐square *p* = 4.06×10^−4^), grade 5 photo‐ageing (Chi‐square *p* = 3.94×10^−3^), and grade 6 photo‐ageing (Chi‐square *p* = 3.48×10^−4^) on the Larnier scale (Figure S7ii).

Similar observations were made using the Griffiths scale. The proportion of participants with grade 0 photo‐ageing on the Griffiths scale and Fitzpatrick Skin Types I through VI are treated as the reference group. Statistically significant comparisons were observed between the reference group and participants with grade 2 photo‐ageing (Chi‐square *p* = 7.23×10^−8^), grade 4 photo‐ageing (Chi‐square *p* = 1.34×10^−4^), grade 6 photo‐ageing (Chi‐square *p* = 3.10×10^−22^), and grade 8 photo‐ageing (Chi‐square *p* = 5.14×10^−3^) (Figure S7i).

Photo‐ageing evaluated using the Korean scale which specialises in the wrinkling constituent of photo‐ageing returned similar results. The proportion of participants with photo 0 photo‐ageing on this photo‐ageing scale and Fitzpatrick Skin Types I through VI are treated as the reference group. Statistically significant comparisons were observed between the reference group and participants with photo 1 photo‐ageing (Chi‐square *p* = 3.04×10^−4^), photo 2 photo‐ageing (Chi‐square *p* = 1.62×10^−5^), photo 3 photo‐ageing (Chi‐square *p* = 1.05×10^−13^), photo 4 photo‐ageing (Chi‐square *p* = 2.75×10^−7^), photo 5 photo‐ageing (Chi‐square *p* = 4.38×10^−9^), and photo 6 photo‐ageing (Chi‐square *p* = 5.91×10^−4^) (Figure S7iii).

Lastly, when photo‐ageing is evaluated using the Korean scale which specialises in the dyspigmentation constituent of photo‐ageing, similar results also emerged. The proportion of participants with photo 0 photo‐ageing on this photo‐ageing scale and Fitzpatrick Skin Types I through VI are treated as the reference group. Statistically significant comparisons were observed between the reference group and participants with photo 1 photo‐ageing (Chi‐square *p* = 1.01×10^−6^), photo 2 photo‐ageing (Chi‐square *p* = 3.76×10^−11^), photo 3 photo‐ageing (Chi‐square *p* = 8.24×10^−7^), photo 4 photo‐ageing (Chi‐square *p* = 5.11×10^−10^), and photo 5 photo‐ageing (Chi‐square *p* = 1.55×10^−5^) (Figure S7iv).

All the Chi‐square *p*‐values in all the above comparisons remain statistically significant after correction for multiple testing through the Bonferroni Correction method.

While a spectrum of skin colours does exist, the way the skin responds to ultraviolet radiation exposure, and therefore photo‐ageing, depends not only on skin colour, but also on whether the skin burns or tans in response to prolonged Sun exposure. The Fitzpatrick Skin Type classification system addresses this diversity and clearly characterises six distinct skin types.

Our data shows that each of the four photo‐ageing scales studied in this paper has incorporated key elements of the six Fitzpatrick Skin Types in their respective scale design process. This is because the proportion of Fitzpatrick Skin Types I through VI in one photo‐numeric grade is significantly different from the proportion of Fitzpatrick Skin Types I through VI in another photo‐numeric grade. Thus, the fact that a wide spectrum of skin colours exists does not appear to complicate the ability of the four scales studied in this paper to grade photo‐ageing effectively.

### 4.4 Areas for future work

Other than photo‐ageing, skin ageing can also be evaluated by assessing facial wrinkles. Validated photo‐numeric Caucasian scales and Chinese scales are available for studying four types of facial wrinkles: Crow's Feet wrinkles, forehead wrinkles, glabellar frowns, and nasolabial folds. Future work will evaluate the performance of both scales on the skin of Singapore and Malaysia ethnic Chinese.

At present, Chinese photo‐ageing scales have yet to be developed. Future work in this area entails comparing newly developed Chinese photo‐ageing scales with the Caucasian scale and Korean scales. The study objective will then be to investigate whether utilising this newly developed Chinese photo‐ageing scale on Chinese skin will enhance our ability to capture wrinkles and dyspigmentation better than the present method of using Caucasian scale and Korean scales.

Another potential area for future work is to integrate skin sagging characteristics into photo‐ageing scales. There are currently no photo‐ageing scales that capture skin sagging, especially sagging in the lower facial region, despite skin sagging being an important age‐related change in the Asians.^15^ The study objective here will then be to develop a photo‐numeric scale that captures the sagging constituent of photo‐ageing. Photo‐numeric scales designed to evaluate skin sagging in the Caucasian,^16^, ^17^ Latin‐American,^11^ and Asian (Japanese) populations^15^ may serve as useful starting points in working towards developing such a scale. When developed, the new scale will also need to be properly validated.

## 5 CONCLUSION

In conclusion, photo‐ageing is a multi‐faceted form of skin ageing. Previous work in the field has enabled photo‐ageing to be evaluated through studying wrinkle variations and dyspigmentation patterns using validated photo‐numeric scales. There are presently no photo‐ageing scales for Chinese skin. Thus, our three trained assessors explored ways to adapt existing Caucasian and Korean photo‐ageing scales to use on ethnic Chinese facial skin. We based our analysis on three sets of independent photo‐ageing assessments. Each assessor evaluated 1,081 ethnic Chinese young adults using both Caucasian scales and Korean scales. All assessors agree that when photo‐ageing is evaluated through studying wrinkle variations, Caucasian scales and Korean scales are strongly concordant and both scales are suitable to evaluate photo‐ageing on the Chinese face. In contrast, the Korean scale that specialises in the dyspigmentation constituent of photo‐ageing provides sufficiently distinct data that cannot be replicated by the Caucasian scale. Taking into consideration background knowledge that Caucasian skin wrinkles more easily and Chinese skin becomes dyspigmented more easily with age, it reasons that the Caucasian scale is better designed to capture wrinkle variations and the Korean scale is better designed to capture dyspigmentation patterns. Until a time when a Chinese scale for photo‐ageing is developed, using a combination of the Caucasian scale to evaluate wrinkle variations and the Korean scale to evaluate dyspigmentation patterns is a satisfactory approximation of photo‐ageing on the ethnic Chinese skin. When developed, the new Chinese scale for photo‐ageing will also need to be properly validated.

## CONFLICT OF INTEREST STATEMENT

F.T.C reports grants from the National University of Singapore, Singapore Ministry of Education Academic Research Fund, Singapore Immunology Network, National Medical Research Council (NMRC) (Singapore), Biomedical Research Council (BMRC) (Singapore), National Research Foundation (NRF) (Singapore), Singapore Food Agency (SFA), and the Agency for Science Technology and Research (A*STAR) (Singapore), during the conduct of the study; and consulting fees from Sime Darby Technology Centre; First Resources Ltd; Genting Plantation, Olam International, and Syngenta Crop Protection, outside the submitted work. The other authors declare no other competing interests.

## CONSENT FOR PUBLICATION

All authors have read and consented to the publication of this manuscript.

## ETHICS APPROVAL AND CONSENT

This study was conducted in accordance with the principles of the Declaration of Helsinki and Good Clinical Practices, and in compliance with local regulatory requirements. The cross‐sectional studies in Singapore were conducted on the National University of Singapore (NUS) campus annually between 2005 and 2023 with the approval of the Institutional Review Board (Reference Code: NUS‐07‐023, NUS‐09‐256, NUS‐10‐445, NUS‐13‐075, NUS‐14‐150, and NUS‐18‐036) and by the Helsinki declaration, of which, participants between 2011 and 2022 participated in the current skin ageing study (Reference Code: NUS‐2020‐495). The cross‐sectional studies in Malaysia were held in Universiti Tunku Abdul Rahman (UTAR) Campus and Sunway University. Ethical approval was granted from the Scientific and Ethical Review Committee of UTAR (Reference Code: U/SERC/03/2016) and the Sunway University Research Ethics Committee (Reference Code: SUREC 2019/029 and SUREC 2022/049). Before the data collection, all participants involved signed an informed consent form.

## Supporting information

Supporting Information

Supporting Information

## Data Availability

All data used and included in this study are available from the corresponding author (F.T.C.).
