# Supplementary material for: Comparisons between Caucasian‐validated photo‐numeric scales and Korean‐validated photo‐numeric scales for photo‐ageing. Insights from the Singapore/Malaysia cross‐sectional genetics epidemiology study (SMCGES) cohort
Source: Skin Res Technol. 2024 May 23;30(5):e13637. doi: 10.1111/srt.13637 (PMC11116842; doi:10.1111/srt.13637)
Supplement: Supplementary file 1 — Supporting Information [file SRT-30-e13637-s001.docx]

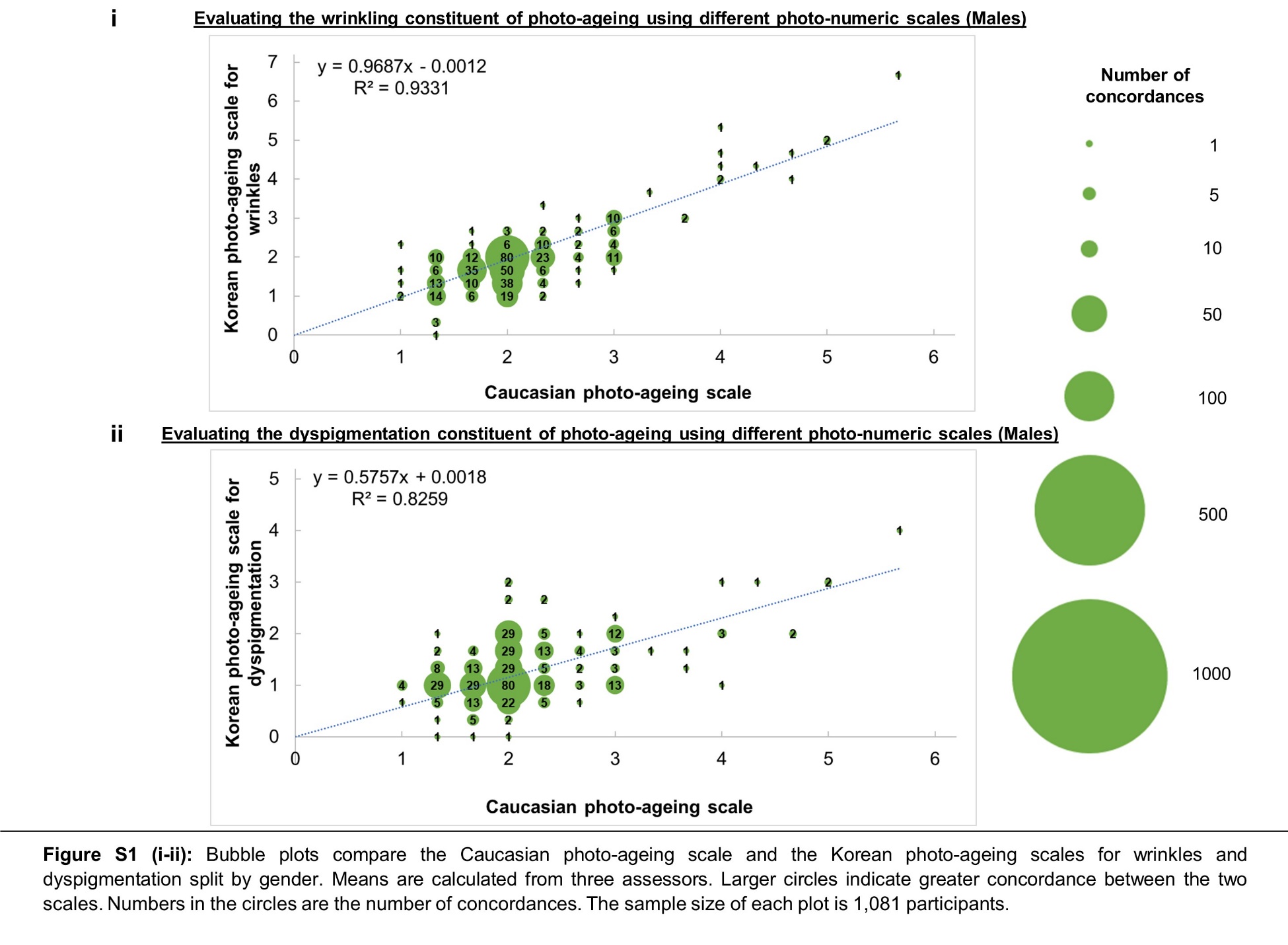


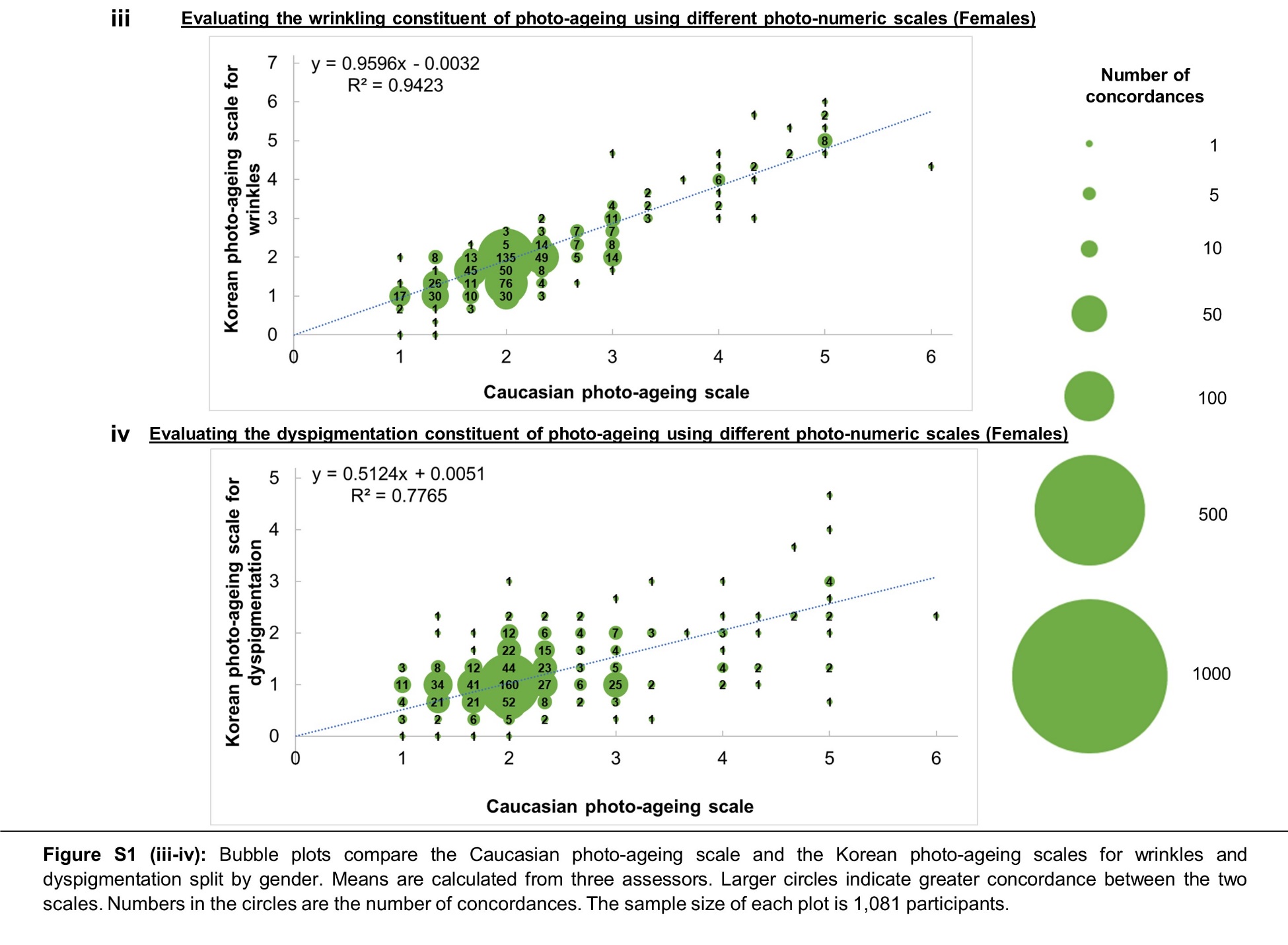


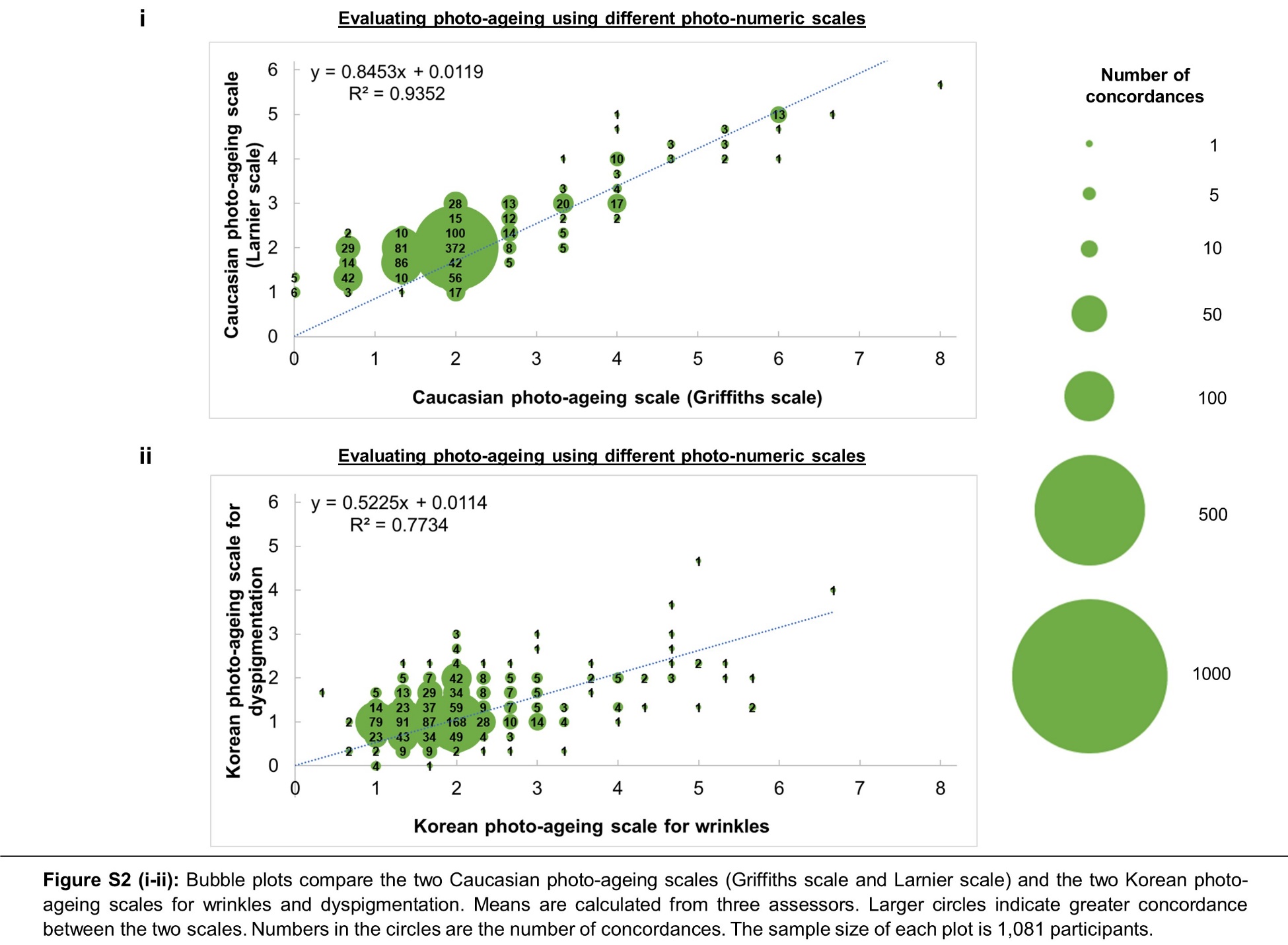


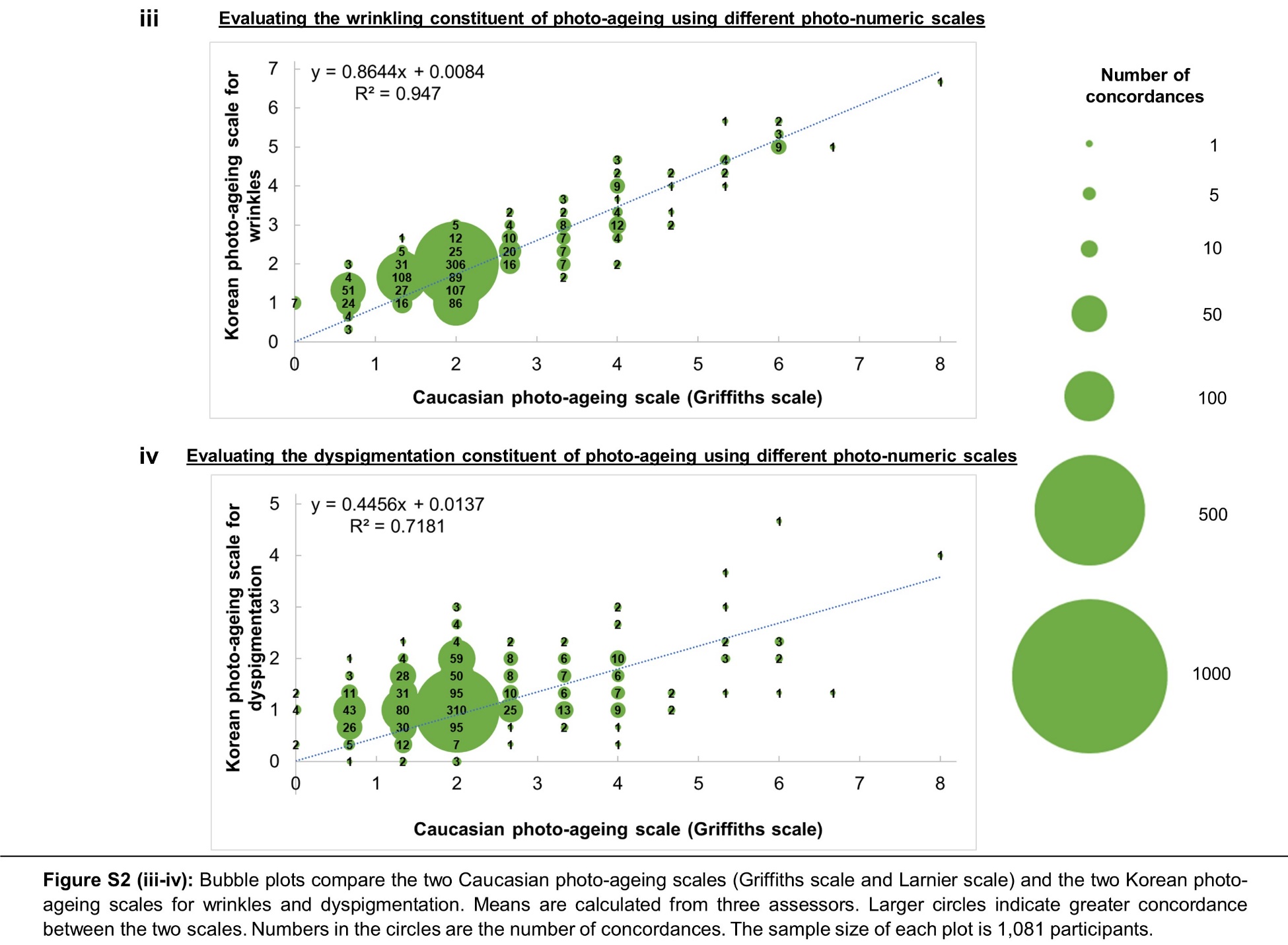


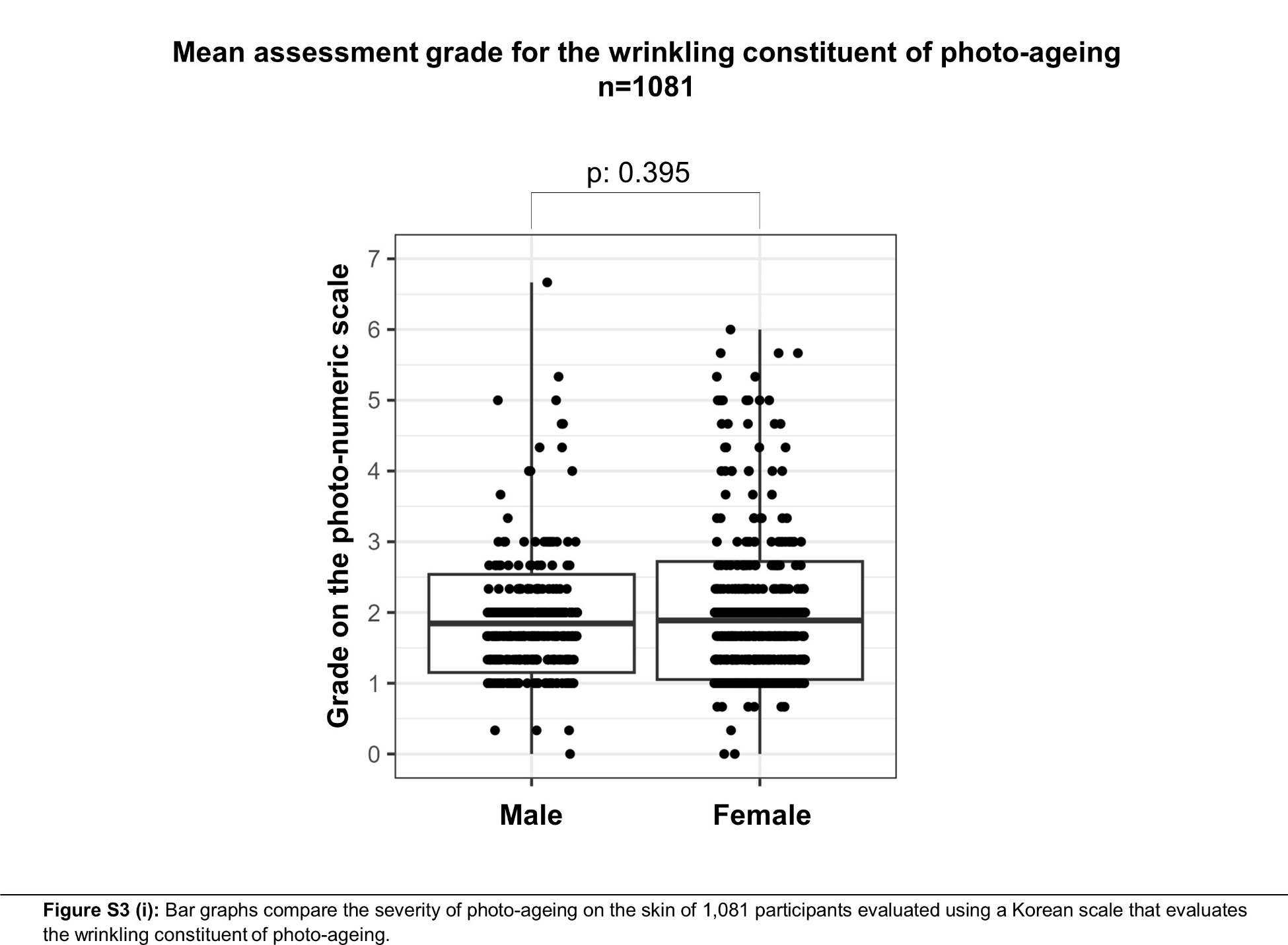


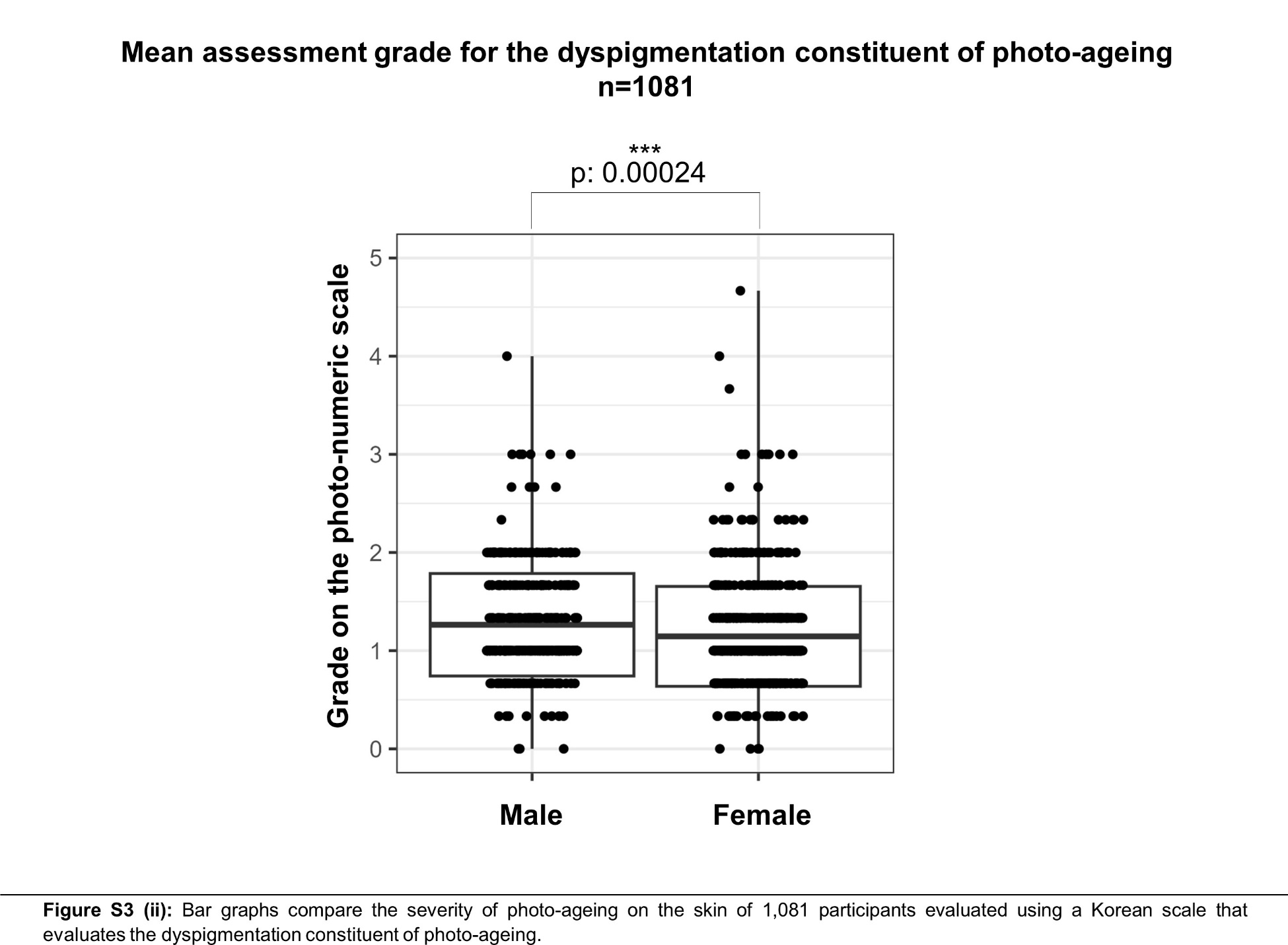


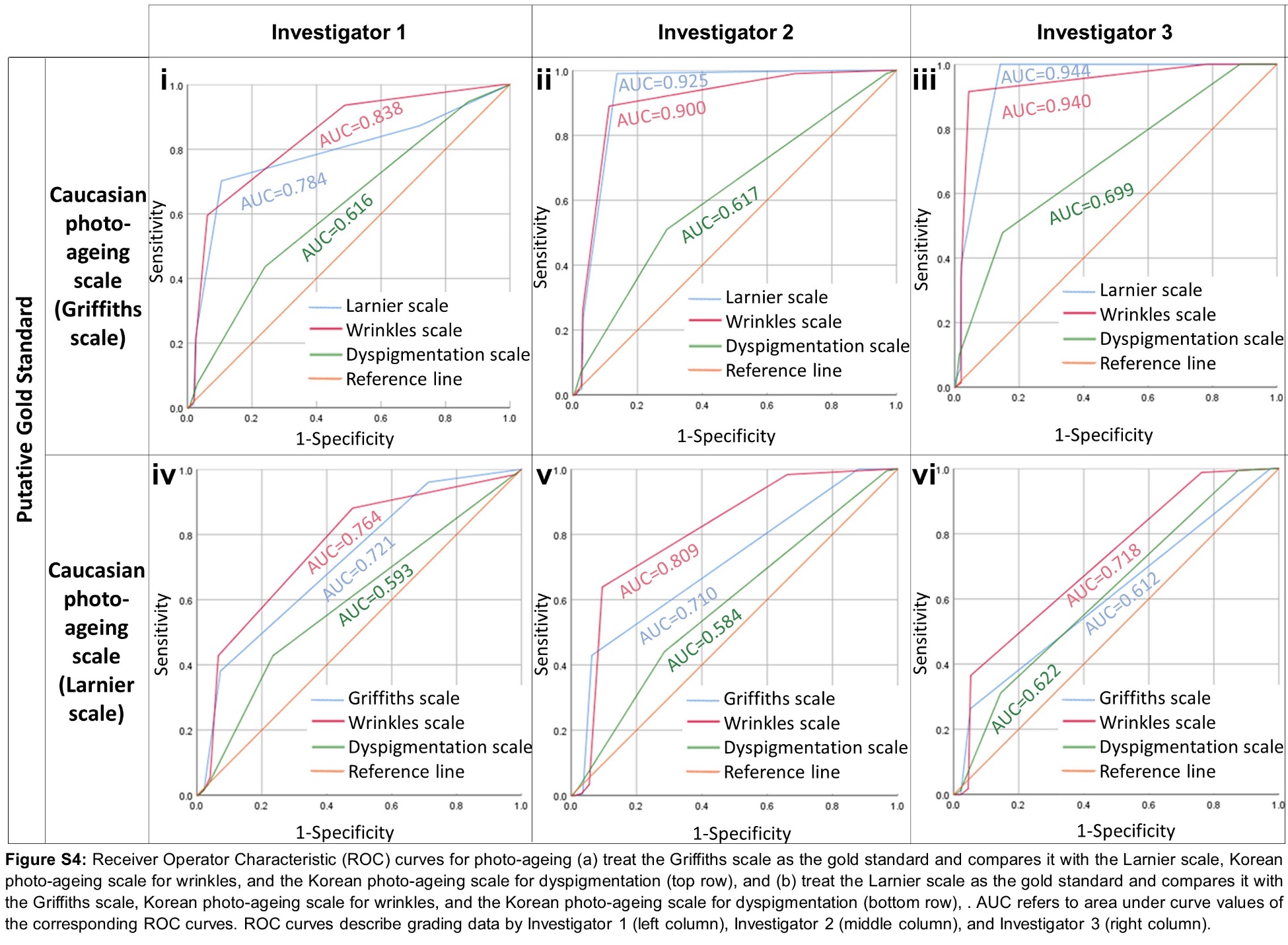


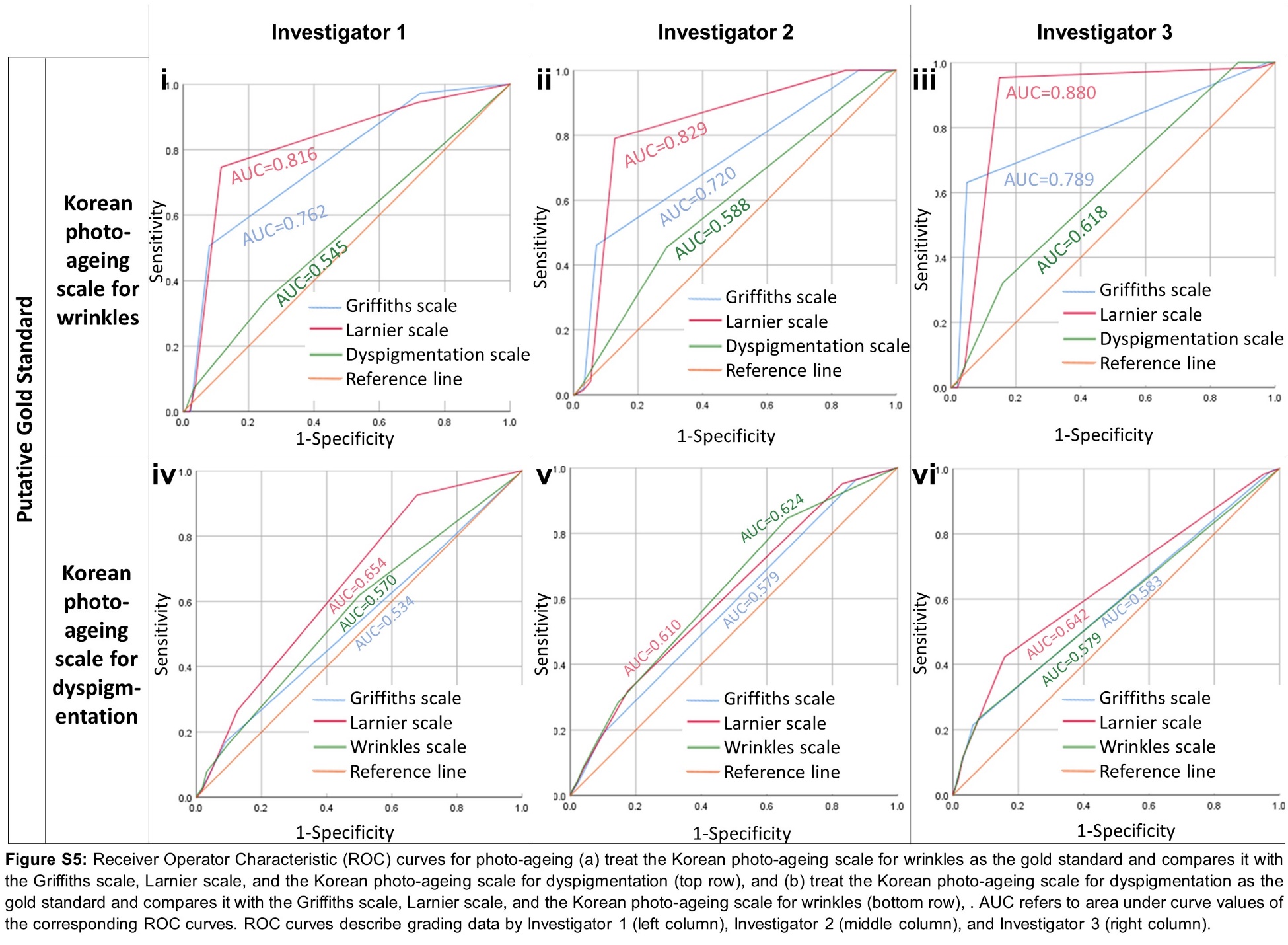


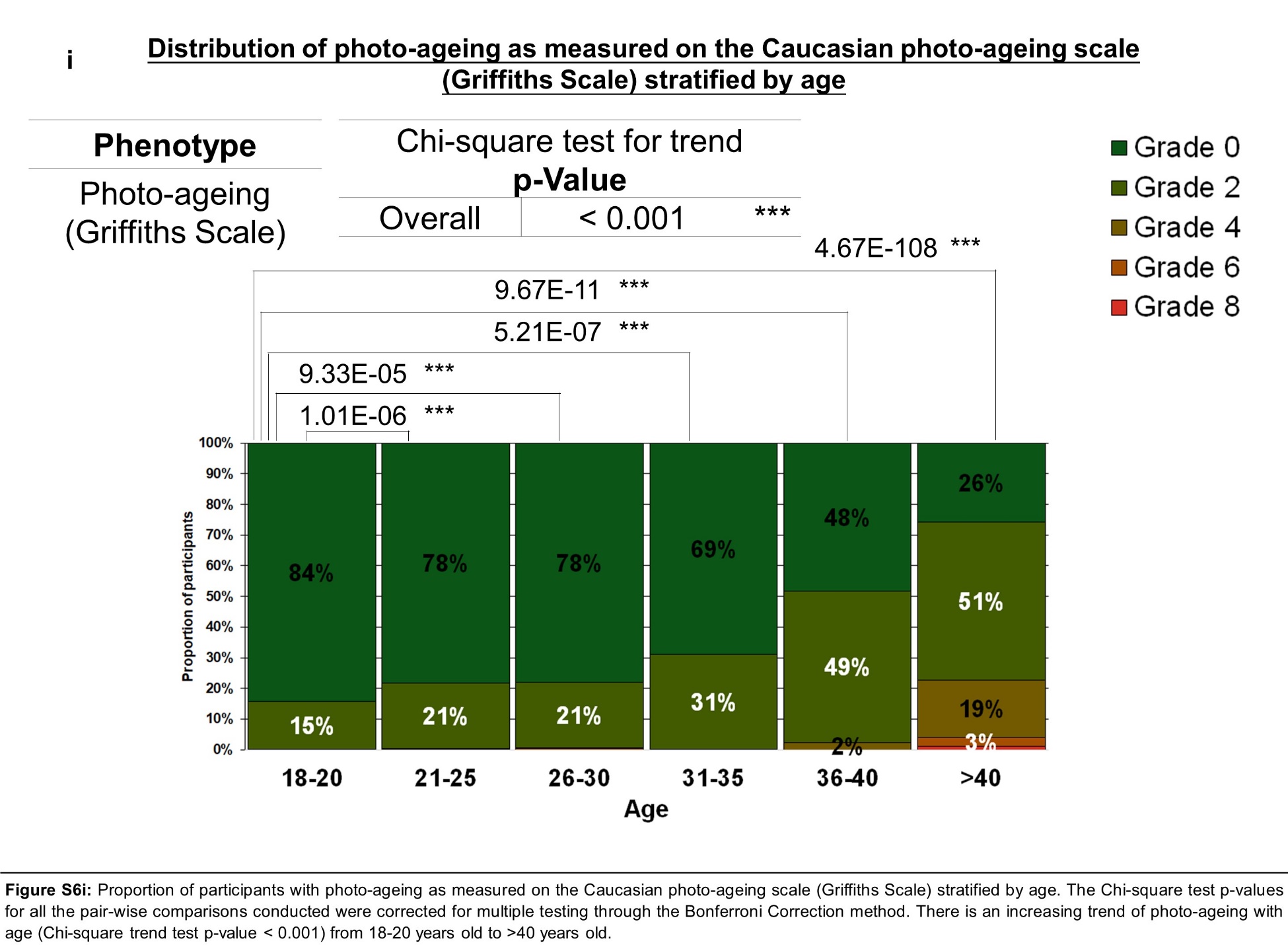


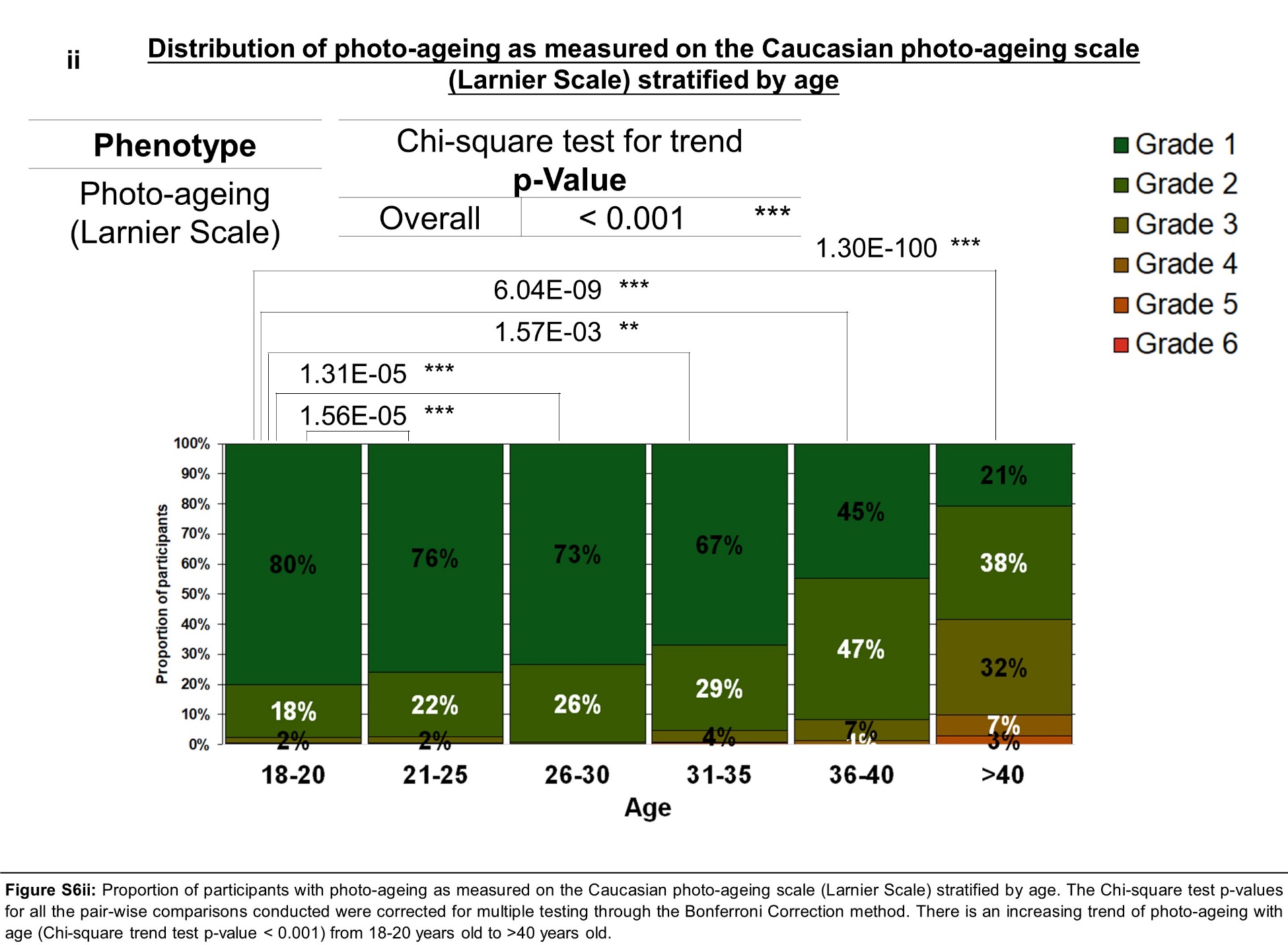


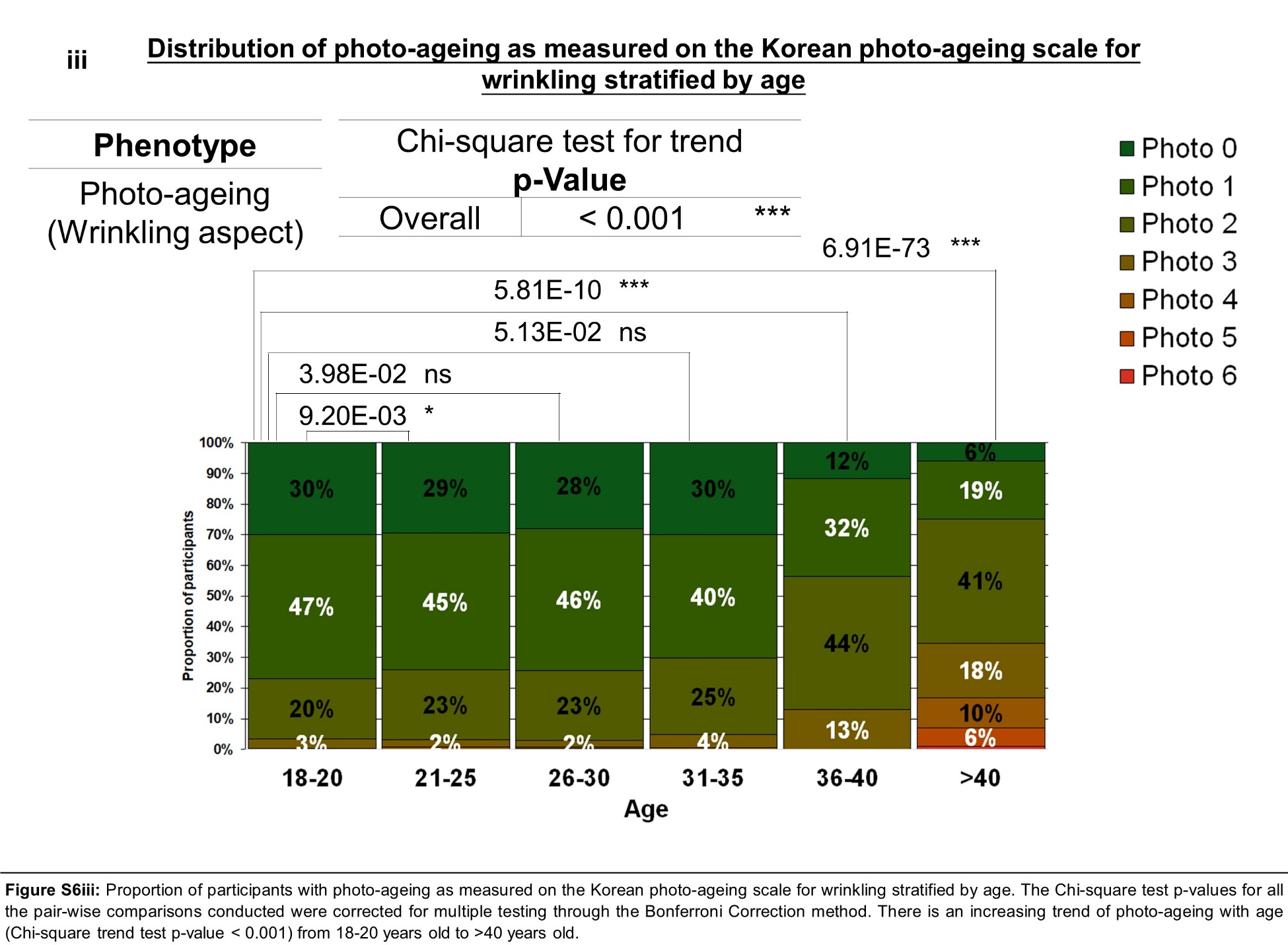


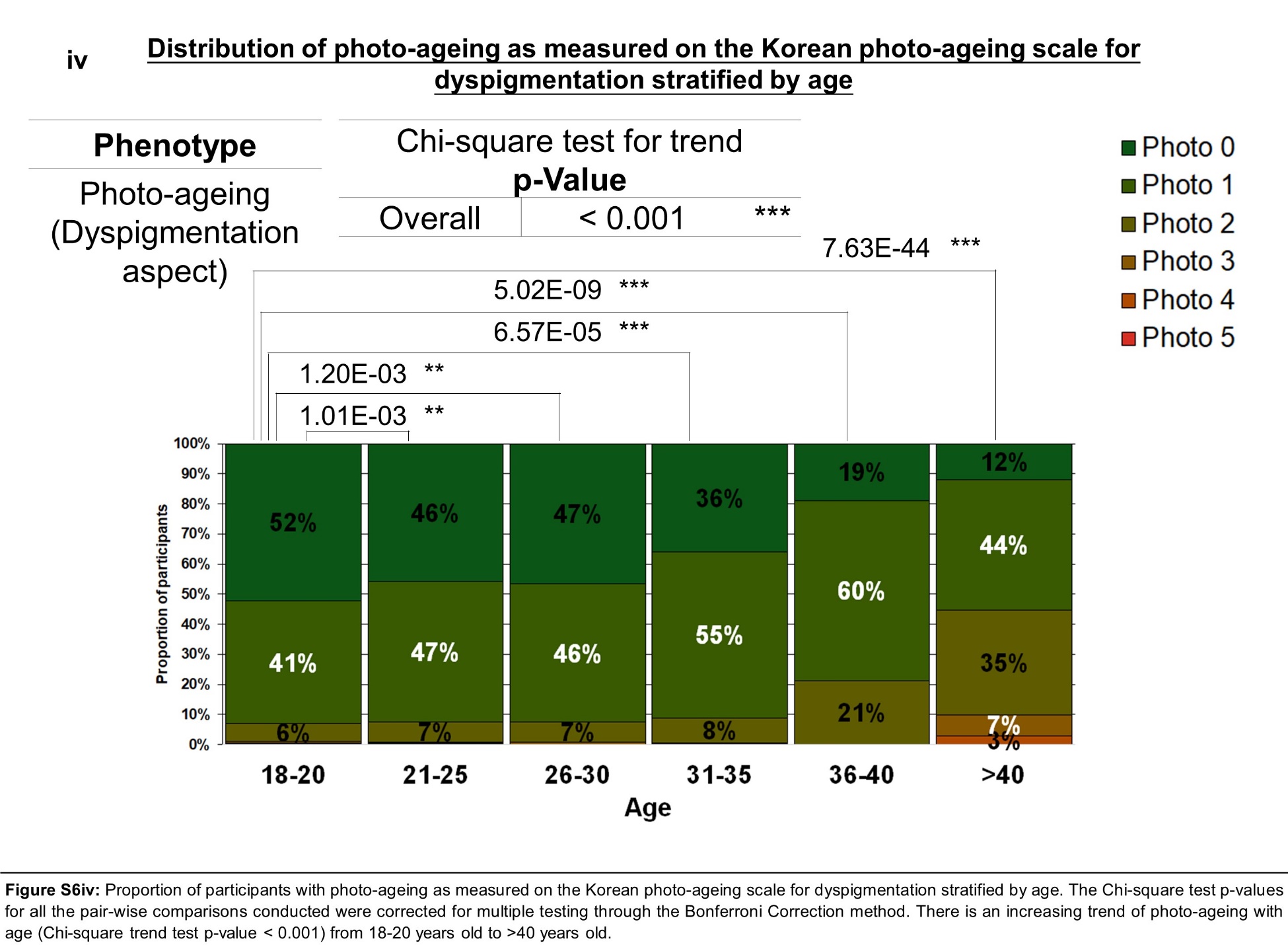


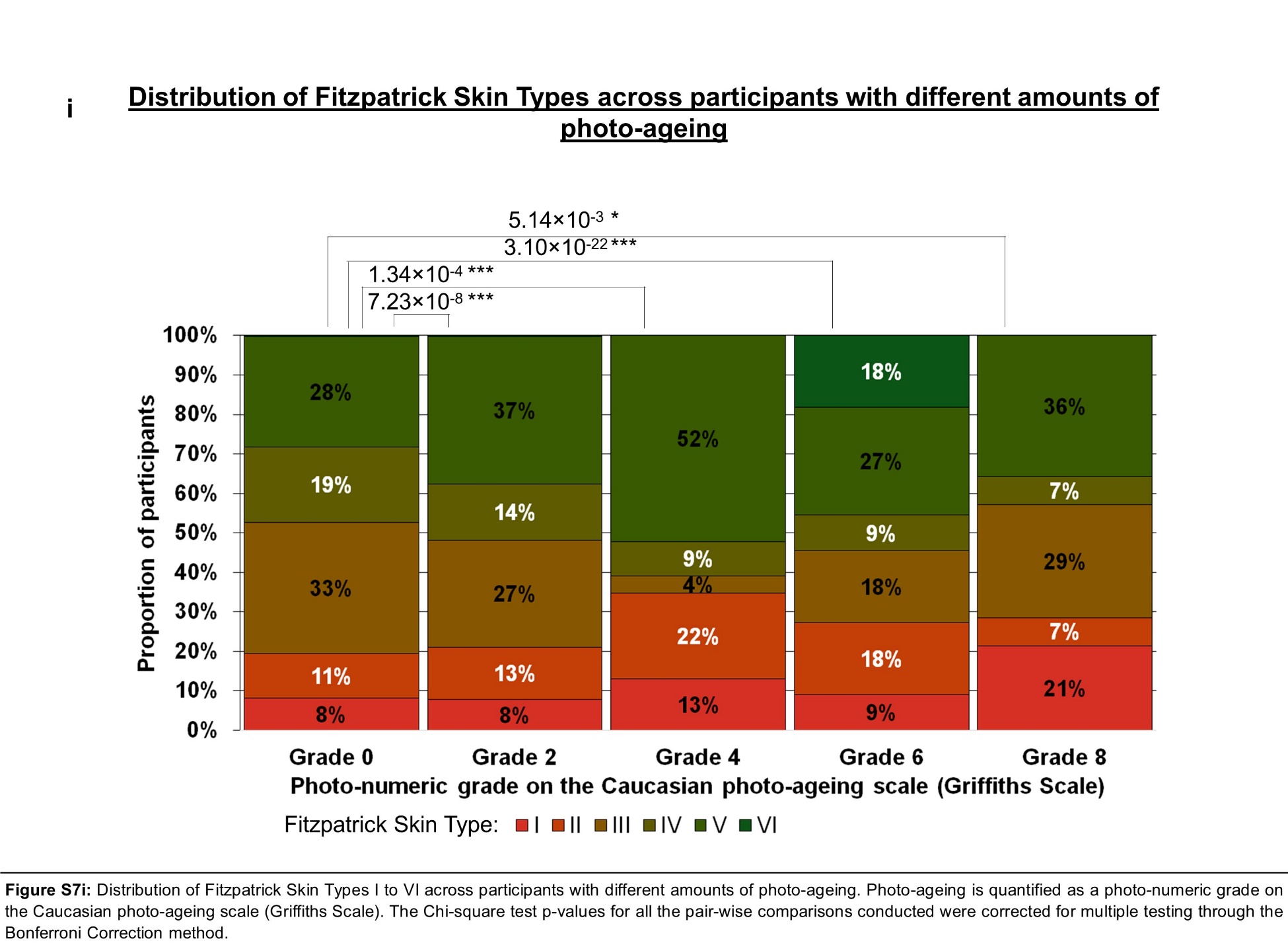


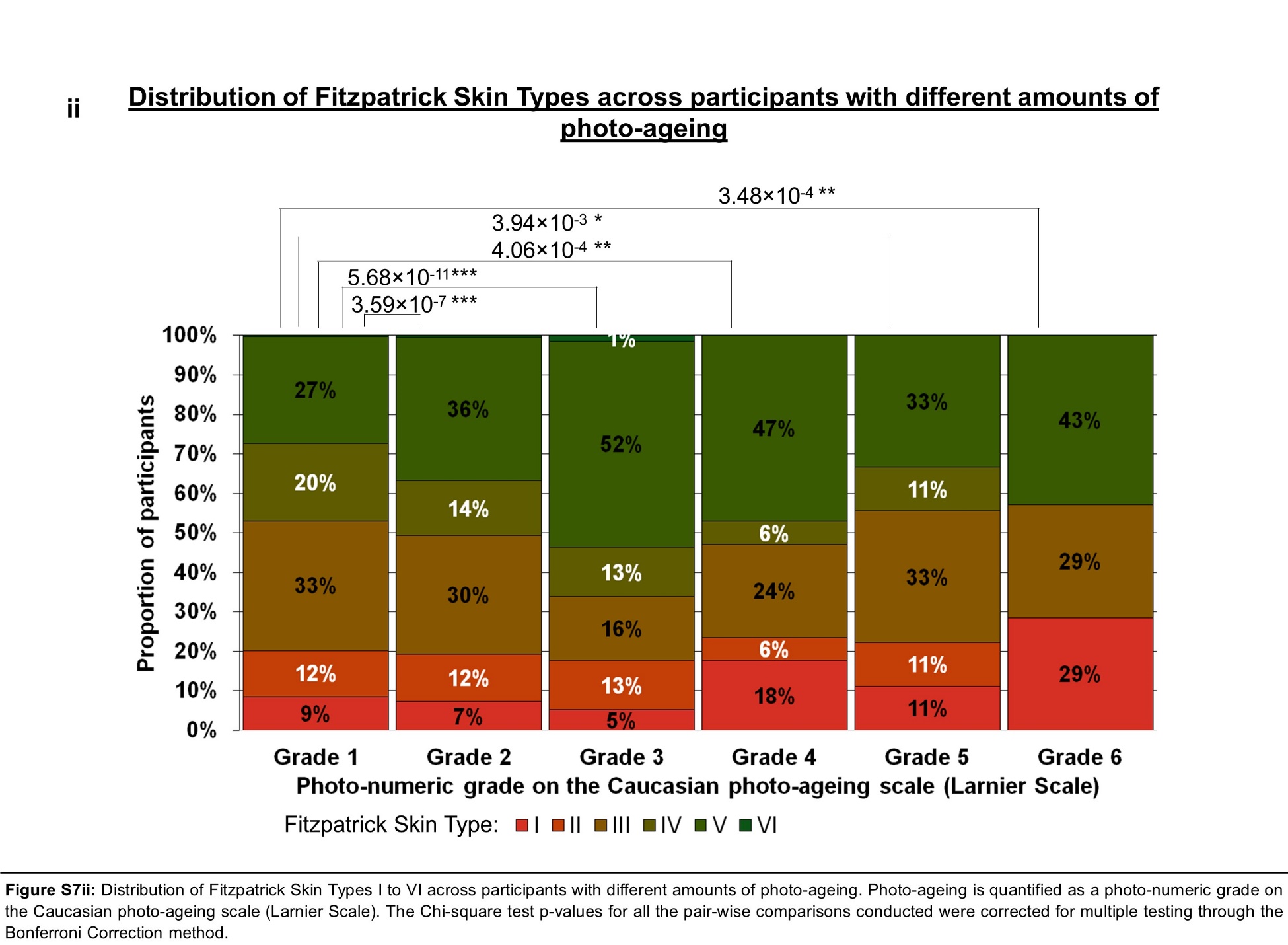


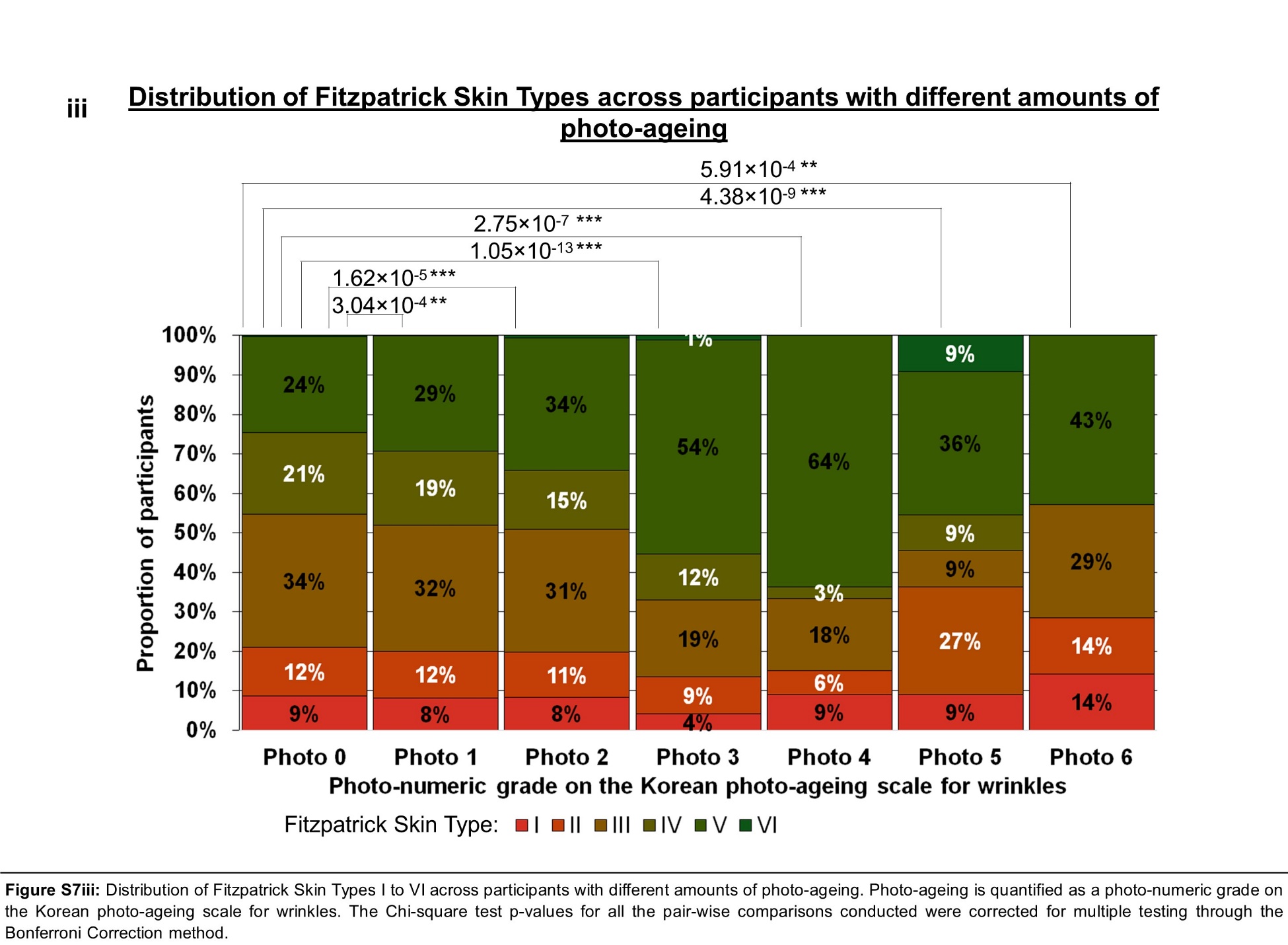


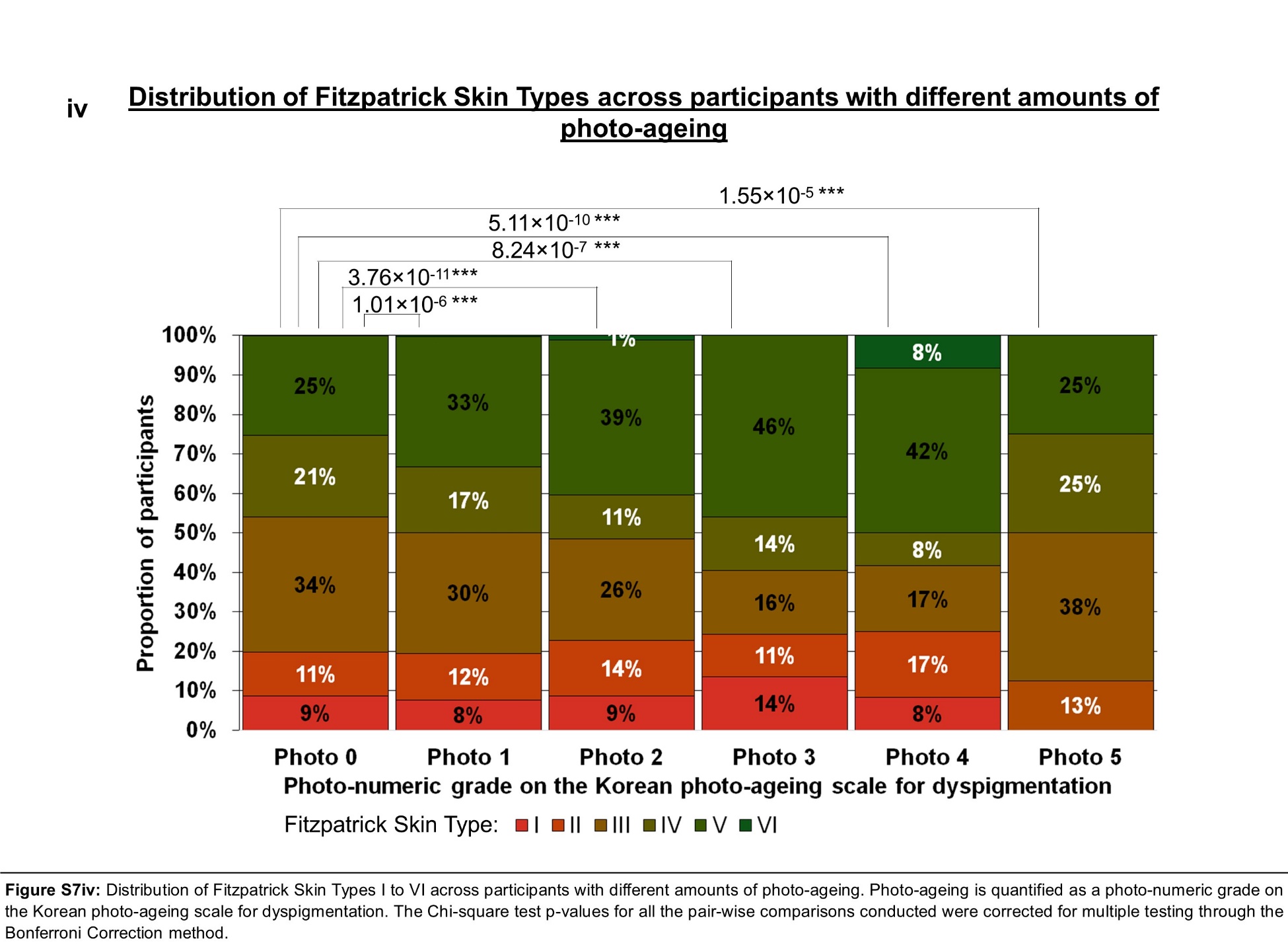


**Supplementary Figure Legends**

**Figure S1i:** Bubble plots compare the Caucasian photo-ageing scale and the Korean photo-ageing scales for wrinkles split by gender. Data from males is shown in this figure. Means are calculated from three assessors. Larger circles indicate greater concordance between the two scales. Numbers in the circles are the number of concordances. The sample size of each plot is 1,081 participants.

**Figure S1ii:** Bubble plots compare the Caucasian photo-ageing scale and the Korean photo-ageing scales for dyspigmentation split by gender. Data from males is shown in this figure. Means are calculated from three assessors. Larger circles indicate greater concordance between the two scales. Numbers in the circles are the number of concordances. The sample size of each plot is 1,081 participants.

**Figure S1iii:** Bubble plots compare the Caucasian photo-ageing scale and the Korean photo-ageing scales for wrinkles split by gender. Data from females is shown in this figure. Means are calculated from three assessors. Larger circles indicate greater concordance between the two scales. Numbers in the circles are the number of concordances. The sample size of each plot is 1,081 participants.

**Figure S1iv:** Bubble plots compare the Caucasian photo-ageing scale and the Korean photo-ageing scales for dyspigmentation split by gender. Data from females is shown in this figure. Means are calculated from three assessors. Larger circles indicate greater concordance between the two scales. Numbers in the circles are the number of concordances. The sample size of each plot is 1,081 participants.

**Figure S2i:** Bubble plots compare the two Caucasian photo-ageing scales (Griffiths scale and Larnier scale). Means are calculated from three assessors. Larger circles indicate greater concordance between the two scales. Numbers in the circles are the number of concordances. The sample size of each plot is 1,081 participants.

**Figure S2ii:** Bubble plots compare the two Korean photo-ageing scales for wrinkles and dyspigmentation. Means are calculated from three assessors. Larger circles indicate greater concordance between the two scales. Numbers in the circles are the number of concordances. The sample size of each plot is 1,081 participants.

**Figure S2iii:** Bubble plots compare a Caucasian photo-ageing scale (Griffiths scale) and a Korean photo-ageing scale for wrinkles. Means are calculated from three assessors. Larger circles indicate greater concordance between the two scales. Numbers in the circles are the number of concordances. The sample size of each plot is 1,081 participants.

**Figure S2iv:** Bubble plots compare a Caucasian photo-ageing scale (Griffiths scale) and a Korean photo-ageing scale for dyspigmentation. Means are calculated from three assessors. Larger circles indicate greater concordance between the two scales. Numbers in the circles are the number of concordances. The sample size of each plot is 1,081 participants.

**Figure S3i:** Bar graphs compare the severity of photo-ageing on the skin of 1,081 participants evaluated using a Korean scale that evaluates the wrinkling constituent of photo-ageing.

**Figure S3ii:** Bar graphs compare the severity of photo-ageing on the skin of 1,081 participants evaluated using a Korean scale that evaluates the dyspigmentation constituent of photo-ageing.

**Figure S4i:** Receiver Operator Characteristic (ROC) curves for photo-ageing treat the Griffiths scale as the gold standard and compares it with the Larnier scale, Korean photo-ageing scale for wrinkles, and the Korean photo-ageing scale for dyspigmentation. AUC refers to area under curve values of the corresponding ROC curves. ROC curves describe grading data by Investigator 1.

**Figure S4ii:** Receiver Operator Characteristic (ROC) curves for photo-ageing treat the Griffiths scale as the gold standard and compares it with the Larnier scale, Korean photo-ageing scale for wrinkles, and the Korean photo-ageing scale for dyspigmentation. AUC refers to area under curve values of the corresponding ROC curves. ROC curves describe grading data by Investigator 2.

**Figure S4iii:** Receiver Operator Characteristic (ROC) curves for photo-ageing treat the Griffiths scale as the gold standard and compares it with the Larnier scale, Korean photo-ageing scale for wrinkles, and the Korean photo-ageing scale for dyspigmentation. AUC refers to area under curve values of the corresponding ROC curves. ROC curves describe grading data by Investigator 3.

**Figure S4iv:** Receiver Operator Characteristic (ROC) curves for photo-ageing treat the Larnier scale as the gold standard and compares it with the Griffiths scale, Korean photo-ageing scale for wrinkles, and the Korean photo-ageing scale for dyspigmentation. AUC refers to area under curve values of the corresponding ROC curves. ROC curves describe grading data by Investigator 1.

**Figure S4v:** Receiver Operator Characteristic (ROC) curves for photo-ageing treat the Larnier scale as the gold standard and compares it with the Griffiths scale, Korean photo-ageing scale for wrinkles, and the Korean photo-ageing scale for dyspigmentation. AUC refers to area under curve values of the corresponding ROC curves. ROC curves describe grading data by Investigator 2.

**Figure S4vi:** Receiver Operator Characteristic (ROC) curves for photo-ageing treat the Larnier scale as the gold standard and compares it with the Griffiths scale, Korean photo-ageing scale for wrinkles, and the Korean photo-ageing scale for dyspigmentation. AUC refers to area under curve values of the corresponding ROC curves. ROC curves describe grading data by Investigator 3.

**Figure S5i:** Receiver Operator Characteristic (ROC) curves for photo-ageing treat the Korean photo-ageing scale for wrinkles as the gold standard and compares it with the Griffiths scale, Larnier scale, and the Korean photo-ageing scale for dyspigmentation. AUC refers to area under curve values of the corresponding ROC curves. ROC curves describe grading data by Investigator 1.

**Figure S5ii:** Receiver Operator Characteristic (ROC) curves for photo-ageing treat the Korean photo-ageing scale for wrinkles as the gold standard and compares it with the Griffiths scale, Larnier scale, and the Korean photo-ageing scale for dyspigmentation. AUC refers to area under curve values of the corresponding ROC curves. ROC curves describe grading data by Investigator 2.

**Figure S5iii:** Receiver Operator Characteristic (ROC) curves for photo-ageing treat the Korean photo-ageing scale for wrinkles as the gold standard and compares it with the Griffiths scale, Larnier scale, and the Korean photo-ageing scale for dyspigmentation. AUC refers to area under curve values of the corresponding ROC curves. ROC curves describe grading data by Investigator 3.

**Figure S5iv:** Receiver Operator Characteristic (ROC) curves for photo-ageing treat the Korean photo-ageing scale for dyspigmentation as the gold standard and compares it with the Griffiths scale, Larnier scale, and the Korean photo-ageing scale for wrinkles. AUC refers to area under curve values of the corresponding ROC curves. ROC curves describe grading data by Investigator 1.

**Figure S5v:** Receiver Operator Characteristic (ROC) curves for photo-ageing treat the Korean photo-ageing scale for dyspigmentation as the gold standard and compares it with the Griffiths scale, Larnier scale, and the Korean photo-ageing scale for wrinkles. AUC refers to area under curve values of the corresponding ROC curves. ROC curves describe grading data by Investigator 2.

**Figure S5vi:** Receiver Operator Characteristic (ROC) curves for photo-ageing treat the Korean photo-ageing scale for dyspigmentation as the gold standard and compares it with the Griffiths scale, Larnier scale, and the Korean photo-ageing scale for wrinkles. AUC refers to area under curve values of the corresponding ROC curves. ROC curves describe grading data by Investigator 3.

**Figure S6i:** Proportion of participants with photo-ageing as measured on the Caucasian photo-ageing scale (Griffiths Scale) stratified by age. The Chi-square test p-values for all the pair-wise comparisons conducted were corrected for multiple testing through the Bonferroni Correction method. There is an increasing trend of photo-ageing with age (Chi-square trend test p-value < 0.001) from 18-20 years old to >40 years old.

**Figure S6ii:** Proportion of participants with photo-ageing as measured on the Caucasian photo-ageing scale (Larnier Scale) stratified by age. The Chi-square test p-values for all the pair-wise comparisons conducted were corrected for multiple testing through the Bonferroni Correction method. There is an increasing trend of photo-ageing with age (Chi-square trend test p-value < 0.001) from 18-20 years old to >40 years old.

**Figure S6iii:** Proportion of participants with photo-ageing as measured on the Korean photo-ageing scale for wrinkling stratified by age. The Chi-square test p-values for all the pair-wise comparisons conducted were corrected for multiple testing through the Bonferroni Correction method. There is an increasing trend of photo-ageing with age (Chi-square trend test p-value < 0.001) from 18-20 years old to >40 years old.

**Figure S6iv:** Proportion of participants with photo-ageing as measured on the Korean photo-ageing scale for dyspigmentation stratified by age. The Chi-square test p-values for all the pair-wise comparisons conducted were corrected for multiple testing through the Bonferroni Correction method. There is an increasing trend of photo-ageing with age (Chi-square trend test p-value < 0.001) from 18-20 years old to >40 years old.

**Figure S7i:** Distribution of Fitzpatrick Skin Types I to VI across participants with different amounts of photo-ageing. Photo-ageing is quantified as a photo-numeric grade on the Caucasian photo-ageing scale (Griffiths Scale). The Chi-square test p-values for all the pair-wise comparisons conducted were corrected for multiple testing through the Bonferroni Correction method.

**Figure S7ii:** Distribution of Fitzpatrick Skin Types I to VI across participants with different amounts of photo-ageing. Photo-ageing is quantified as a photo-numeric grade on the Caucasian photo-ageing scale (Larnier Scale). The Chi-square test p-values for all the pair-wise comparisons conducted were corrected for multiple testing through the Bonferroni Correction method.

**Figure S7iii:** Distribution of Fitzpatrick Skin Types I to VI across participants with different amounts of photo-ageing. Photo-ageing is quantified as a photo-numeric grade on the Korean photo-ageing scale for wrinkles. The Chi-square test p-values for all the pair-wise comparisons conducted were corrected for multiple testing through the Bonferroni Correction method.

**Figure S7iv:** Distribution of Fitzpatrick Skin Types I to VI across participants with different amounts of photo-ageing. Photo-ageing is quantified as a photo-numeric grade on the Korean photo-ageing scale for dyspigmentation. The Chi-square test p-values for all the pair-wise comparisons conducted were corrected for multiple testing through the Bonferroni Correction method.
