# Supplementary material for: Comparisons between Caucasian‐validated photo‐numeric scales and Korean‐validated photo‐numeric scales for photo‐ageing. Insights from the Singapore/Malaysia cross‐sectional genetics epidemiology study (SMCGES) cohort
Source: Skin Res Technol. 2024 May 23;30(5):e13637. doi: 10.1111/srt.13637 (PMC11116842; doi:10.1111/srt.13637)
Supplement: Supplementary file 2 — Supporting Information [file SRT-30-e13637-s002.docx]

**Table S1**: Sources of the validated photo-numeric scales.

| **Name of the validated photo-numeric scale designed to measure photo-ageing** | **Title of the paper** | **Citation of the paper** |
| --- | --- | --- |
| Photo-ageing as measured by the Griffiths scale | A Photonumeric Scale for the Assessment of Cutaneous Photodamage. | Griffiths, C.E.M., Wang, T.S., Hamilton, T.A., Voorhees, J.J., and Ellis, C.N. (1992). A Photonumeric Scale for the Assessment of Cutaneous Photodamage. Arch. Dermatol. *128*, 1406. |
| Photo-ageing as measured by the Larnier scale | Evaluation of cutaneous photodamage using a photographic scale. | Larnier, C., Ortonne, J. ‐P, Venot, A., Faivre, B., Béani, J. ‐C, Thomas, P., Brown, T.C., and Sendagorta, E. (1994). Evaluation of cutaneous photodamage using a photographic scale. Br. J. Dermatol. 130, 167–173. |
| Photo-ageing as measured by the Korean scale (wrinkling constituent) | Cutaneous photodamage in Asians. | Jin Ho, C., Seong Hun, L., Choon Shik, Y., Byung Joo, P., Kyu Han, K., Kyung Chan, P., Kwang Hyun, C., and Hee Chul, E. (2001). Cutaneous photodamage in Asians. J. Dermatol. *28*, 614–616. |
| Photo-ageing as measured by the Korean scale (dyspigmentation constituent) | Cutaneous photodamage in Asians. | Jin Ho, C., Seong Hun, L., Choon Shik, Y., Byung Joo, P., Kyu Han, K., Kyung Chan, P., Kwang Hyun, C., and Hee Chul, E. (2001). Cutaneous photodamage in Asians. J. Dermatol. *28*, 614–616. |

**Table S2**: Comparison between the Caucasian (Griffiths) photo-numeric scale and the Caucasian (Larnier) photo-numeric scale for assessing photo-ageing. Means are calculated from three assessors.

| Measurement | | Phenotype |
| --- | --- | --- |
|  |  | **Photo-ageing** |
| Mean Spearman’s Rank Correlation (ρ) | Value | 0.61 ± 0.08 |
|  | p-Value | 8.03E-79 |
| Mean Cohen’s Kappa (κ) | Value | 0.40 ± 0.04 |
|  | p-Value | 4.70E-65 |
| Mean area under curve (AUC) of the Receiver Operator Characteristic (ROC) curve | When the Caucasian (Griffiths) photo-numeric scale is the gold standard | 0.87 ± 0.01 |
|  | When the Caucasian (Larnier) photo-numeric scale is the gold standard | 0.69 ± 0.01 |
| Mean Coefficient of determination (R^2^) for the equation for the goodness of fit | | 0.9352 |
| Equation for the goodness of fit | | Larnier scale = 0.8453 * Griffiths scale + 0.0119 |

**Table S3**: Inter-assessor concordance, agreement, sensitivity, specificity, and area under curve (AUC) used for calibration.

| **Comparison** | | | **Statistical analysis of the grades given by each of the three assessors before the calibration exercise (n=30)** | | | | | | | | | | | |
| --- | --- | --- | --- | --- | --- | --- | --- | --- | --- | --- | --- | --- | --- | --- |
| **Putative Gold Standard** | | | **Investigator 1** | | | | **Investigator 2** | | | | **Investigator 3** | | | |
| **Pairs involved in the comparison** | | | Investigator 1 vs Investigator 2 | | Investigator 1 vs Investigator 3 | | Investigator 2 vs Investigator 1 | | Investigator 2 vs Investigator 3 | | Investigator 3 vs Investigator 1 | | Investigator 3 vs Investigator 2 | |
| **Phenotype** | **Photo-numeric scale investigated** | **Test** | Value | p-value | Value | p-value | Value | p-value | Value | p-value | Value | p-value | Value | p-value |
| **Photo-ageing** | Photo-ageing as measured by the Griffiths scale | Pearson's R | 0.668 | **2.20E-140** | 0.605 | **7.58E-109** | 0.668 | **2.20E-140** | 0.647 | **2.48E-129** | 0.605 | **7.58E-109** | 0.647 | **2.48E-129** |
|  |  | Spearman's Rank Correlation (ρ) | 0.591 | **1.15E-102** | 0.495 | **4.98E-68** | 0.591 | **1.15E-102** | 0.532 | **3.60E-80** | 0.495 | **4.98E-68** | 0.532 | **3.60E-80** |
|  |  | Cohen’s Kappa (κ) | 0.465 | **5.00E-117** | 0.287 | **1.85E-80** | 0.465 | **5.00E-117** | 0.374 | **2.25E-82** | 0.287 | **1.85E-80** | 0.374 | **2.25E-82** |
|  |  | Sensitivity | 98.002 |  | 99.750 |  | 80.761 |  | 98.868 |  | 75.520 |  | 90.832 |  |
|  |  | Specificity | 33.214 |  | 7.500 |  | 85.321 |  | 11.009 |  | 91.304 |  | 52.174 |  |
|  |  | Area under curve (AUC) | 0.846 | **1.20E-27** | 0.770 | **2.15E-17** | 0.782 | **1.29E-20** | 0.683 | **1.67E-09** | 0.870 | **1.86E-25** | 0.847 | **1.30E-22** |
|  | Photo-ageing as measured by the Larnier scale | Pearson's R | 0.731 | **4.41E-181** | 0.700 | **6.98E-160** | 0.731 | **4.41E-181** | 0.728 | **1.89E-179** | 0.700 | **6.98E-160** | 0.728 | **1.89E-179** |
|  |  | Spearman's Rank Correlation (ρ) | 0.610 | **4.14E-111** | 0.548 | **1.18E-85** | 0.610 | **4.14E-111** | 0.573 | **2.29E-95** | 0.548 | **1.18E-85** | 0.573 | **2.29E-95** |
|  |  | Cohen’s Kappa (κ) | 0.494 | **5.53E-139** | 0.336 | **6.34E-83** | 0.494 | **5.53E-139** | 0.379 | **3.30E-80** | 0.336 | **6.34E-83** | 0.379 | **3.30E-80** |
|  |  | Sensitivity | 97.704 |  | 98.980 |  | 81.925 |  | 97.647 |  | 75.048 |  | 88.298 |  |
|  |  | Specificity | 43.098 |  | 13.131 |  | 87.671 |  | 17.123 |  | 82.979 |  | 53.191 |  |
|  |  | Area under curve (AUC) | 0.808 | **2.30E-28** | 0.799 | **7.40E-27** | 0.682 | **2.68E-14** | 0.691 | **1.33E-15** | 0.745 | **4.11E-24** | 0.743 | **1.13E-23** |
|  | Photo-ageing as measured by the Korean scale (wrinkles constituent) | Pearson's R | 0.708 | **6.69E-165** | 0.664 | **2.89E-138** | 0.708 | **6.69E-165** | 0.688 | **2.81E-152** | 0.664 | **2.89E-138** | 0.688 | **2.81E-152** |
|  |  | Spearman's Rank Correlation (ρ) | 0.553 | **1.27E-87** | 0.488 | **8.18E-66** | 0.553 | **1.27E-87** | 0.516 | **1.11E-74** | 0.488 | **8.18E-66** | 0.516 | **1.11E-74** |
|  |  | Cohen’s Kappa (κ) | 0.439 | **2.68E-112** | 0.316 | **4.24E-64** | 0.439 | **2.68E-112** | 0.298 | **4.55E-51** | 0.316 | **4.24E-64** | 0.298 | **4.55E-51** |
|  |  | Sensitivity | 99.327 |  | 99.519 |  | 96.813 |  | 99.531 |  | 96.369 |  | 98.883 |  |
|  |  | Specificity | 17.073 |  | 4.878 |  | 50.000 |  | 14.286 |  | 28.571 |  | 28.571 |  |
|  |  | Area under curve (AUC) | 0.872 | **3.65E-24** | 0.771 | **1.41E-13** | 0.632 | **4.05E-07** | 0.647 | **1.61E-08** | 0.828 | **2.08E-19** | 0.829 | **1.83E-19** |
|  | Photo-ageing as measured by the Korean scale (dyspigmentation constituent) | Pearson's R | 0.513 | **1.03E-73** | 0.360 | **2.13E-34** | 0.513 | **1.03E-73** | 0.437 | **1.25E-51** | 0.360 | **2.13E-34** | 0.437 | **1.25E-51** |
|  |  | Spearman's Rank Correlation (ρ) | 0.492 | **7.65E-67** | 0.294 | **5.73E-23** | 0.492 | **7.65E-67** | 0.368 | **5.47E-36** | 0.294 | **5.73E-23** | 0.368 | **5.47E-36** |
|  |  | Cohen’s Kappa (κ) | 0.414 | **5.75E-79** | 0.236 | **2.25E-29** | 0.414 | **5.75E-79** | 0.196 | **6.91E-20** | 0.236 | **2.25E-29** | 0.196 | **6.91E-20** |
|  |  | Sensitivity | 97.992 |  | 88.689 |  | 88.286 |  | 89.333 |  | 87.670 |  | 98.015 |  |
|  |  | Specificity | 8.889 |  | 12.593 |  | 38.710 |  | 38.710 |  | 13.710 |  | 9.677 |  |
|  |  | Area under curve (AUC) | 0.766 | **6.74E-36** | 0.648 | **2.71E-12** | 0.728 | **1.68E-31** | 0.623 | **2.72E-10** | 0.691 | **1.77E-14** | 0.695 | **4.68E-15** |

**Table S4**: Inter-assessor concordance, agreement, sensitivity, specificity, and area under curve (AUC) after calibration.

| **Comparison** | | | **Statistical analysis of the grades given by each of the three assessors before the calibration exercise (n=30)** | | | | | | | | | | | |
| --- | --- | --- | --- | --- | --- | --- | --- | --- | --- | --- | --- | --- | --- | --- |
| **Putative Gold Standard** | | | **Investigator 1** | | | | **Investigator 2** | | | | **Investigator 3** | | | |
| **Pairs involved in the comparison** | | | Investigator 1 vs Investigator 2 | | Investigator 1 vs Investigator 3 | | Investigator 2 vs Investigator 1 | | Investigator 2 vs Investigator 3 | | Investigator 3 vs Investigator 1 | | Investigator 3 vs Investigator 2 | |
| **Phenotype** | **Photo-numeric scale investigated** | **Test** | Value | p-value | Value | p-value | Value | p-value | Value | p-value | Value | p-value | Value | p-value |
| **Photo-ageing** | Photo-ageing as measured by the Griffiths scale | Pearson's R | 0.680 | **2.61E-147** | 0.618 | **4.24E-115** | 0.680 | **2.61E-147** | 0.647 | **2.48E-129** | 0.618 | **4.24E-115** | 0.647 | **2.48E-129** |
|  |  | Spearman's Rank Correlation (ρ) | 0.598 | **9.63E-106** | 0.506 | **2.85E-71** | 0.598 | **9.63E-106** | 0.532 | **3.60E-80** | 0.506 | **2.85E-71** | 0.532 | **3.60E-80** |
|  |  | Cohen’s Kappa (κ) | 0.467 | **1.92E-117** | 0.289 | **7.73E-81** | 0.467 | **1.92E-117** | 0.374 | **2.25E-82** | 0.289 | **7.73E-81** | 0.374 | **2.25E-82** |
|  |  | Sensitivity | 98.005 |  | 99.751 |  | 80.864 |  | 98.868 |  | 75.614 |  | 90.832 |  |
|  |  | Specificity | 33.333 |  | 7.527 |  | 85.321 |  | 11.009 |  | 91.304 |  | 52.174 |  |
|  |  | Area under curve (AUC) | 0.838 | **2.50E-27** | 0.763 | **2.86E-17** | 0.763 | **2.86E-17** | 0.791 | **7.33E-22** | 0.683 | **1.67E-09** | 0.882 | **3.97E-27** |
|  | Photo-ageing as measured by the Larnier scale | Pearson's R | 0.764 | **1.47E-207** | 0.737 | **6.32E-186** | 0.764 | **1.47E-207** | 0.738 | **2.05E-186** | 0.737 | **6.32E-186** | 0.738 | **2.05E-186** |
|  |  | Spearman's Rank Correlation (ρ) | 0.644 | **1.39E-127** | 0.598 | **8.15E-106** | 0.644 | **1.39E-127** | 0.584 | **5.48E-100** | 0.598 | **8.15E-106** | 0.584 | **5.48E-100** |
|  |  | Cohen’s Kappa (κ) | 0.505 | **1.97E-144** | 0.346 | **2.01E-86** | 0.505 | **1.97E-144** | 0.386 | **6.22E-83** | 0.346 | **2.01E-86** | 0.386 | **6.22E-83** |
|  |  | Sensitivity | 97.727 |  | 98.990 |  | 82.781 |  | 97.647 |  | 75.969 |  | 88.469 |  |
|  |  | Specificity | 44.291 |  | 14.187 |  | 87.671 |  | 18.493 |  | 83.673 |  | 55.102 |  |
|  |  | Area under curve (AUC) | 0.813 | **2.86E-30** | 0.808 | **2.65E-29** | 0.711 | **5.43E-19** | 0.696 | **1.73E-16** | 0.780 | **1.01E-30** | 0.750 | **7.65E-25** |
|  | Photo-ageing as measured by the Korean scale (wrinkles constituent) | Pearson's R | 0.771 | **7.83E-214** | 0.733 | **6.76E-183** | 0.771 | **7.83E-214** | 0.724 | **1.62E-176** | 0.733 | **6.76E-183** | 0.724 | **1.62E-176** |
|  |  | Spearman's Rank Correlation (ρ) | 0.609 | **7.13E-111** | 0.543 | **4.45E-84** | 0.609 | **7.13E-111** | 0.548 | **6.51E-86** | 0.543 | **4.45E-84** | 0.548 | **6.51E-86** |
|  |  | Cohen’s Kappa (κ) | 0.453 | **3.42E-113** | 0.331 | **1.36E-66** | 0.453 | **3.42E-113** | 0.317 | **4.00E-57** | 0.331 | **1.36E-66** | 0.317 | **4.00E-57** |
|  |  | Sensitivity | 99.341 |  | 99.529 |  | 98.875 |  | 99.531 |  | 98.509 |  | 98.975 |  |
|  |  | Specificity | 36.842 |  | 15.789 |  | 50.000 |  | 21.429 |  | 37.500 |  | 37.500 |  |
|  |  | Area under curve (AUC) | 0.866 | **5.67E-25** | 0.789 | **4.08E-16** | 0.673 | **2.55E-11** | 0.674 | **1.95E-11** | 0.855 | **7.50E-22** | 0.833 | **2.21E-19** |
|  | Photo-ageing as measured by the Korean scale (dyspigmentation constituent) | Pearson's R | 0.603 | **4.08E-108** | 0.448 | **1.33E-54** | 0.603 | **4.08E-108** | 0.518 | **4.20E-75** | 0.448 | **1.33E-54** | 0.518 | **4.20E-75** |
|  |  | Spearman's Rank Correlation (ρ) | 0.555 | **2.48E-88** | 0.379 | **2.62E-38** | 0.555 | **2.48E-88** | 0.431 | **4.95E-50** | 0.379 | **2.62E-38** | 0.431 | **4.95E-50** |
|  |  | Cohen’s Kappa (κ) | 0.414 | **5.75E-79** | 0.236 | **2.25E-29** | 0.414 | **5.75E-79** | 0.196 | **6.91E-20** | 0.236 | **2.25E-29** | 0.196 | **6.91E-20** |
|  |  | Sensitivity | 97.992 |  | 88.689 |  | 88.286 |  | 89.333 |  | 87.670 |  | 98.015 |  |
|  |  | Specificity | 8.889 |  | 12.593 |  | 38.710 |  | 38.710 |  | 13.710 |  | 9.677 |  |
|  |  | Area under curve (AUC) | 0.766 | **6.74E-36** | 0.648 | **2.71E-12** | 0.728 | **1.68E-31** | 0.623 | **2.72E-10** | 0.691 | **1.77E-14** | 0.695 | **4.68E-15** |

**Table S5**: Weighted kappa coefficient for intra-assessor agreement.

| **Photo-numeric scale investigated** | **Assessor Number** | **Kappa** | **Weighted Kappa** | | |
| --- | --- | --- | --- | --- | --- |
|  |  |  | **κ_w_** | **95% CI** | |
| Photo-ageing as measured by the Griffiths scale | 1 | 0.993 | 0.993 | 0.985 | 1.000 |
|  | 2 | 1.000 | 1.000 | 1.000 | 1.000 |
|  | 3 | 1.000 | 1.000 | 1.000 | 1.000 |
| Photo-ageing as measured by the Larnier scale | 1 | 0.983 | 0.980 | 0.967 | 0.992 |
|  | 2 | 0.995 | 0.994 | 0.986 | 1.001 |
|  | 3 | 0.995 | 0.993 | 0.983 | 1.003 |
| Photo-ageing as measured by the Korean scale (wrinkles constituent) | 1 | 0.952 | 0.952 | 0.934 | 0.969 |
|  | 2 | 0.988 | 0.984 | 0.972 | 0.995 |
|  | 3 | 0.980 | 0.978 | 0.964 | 0.992 |
| Photo-ageing as measured by the Korean scale (dyspigmentation constituent) | 1 | 0.973 | 0.966 | 0.949 | 0.984 |
|  | 2 | 0.981 | 0.968 | 0.949 | 0.988 |
|  | 3 | 0.973 | 0.968 | 0.950 | 0.986 |

**Table S6**: Comparison among the (i) Griffiths scale, (ii) Larnier scale, (iii) Korean scale (wrinkles constituent), and (iv) Korean scale (dyspigmentation constituent) for assessing photo-ageing. The Korean scales are photo-numeric scales developed on Korean skin for assessing the wrinkling and dyspigmentation constituents of photo-ageing. The table reports grading data split by assessor.

| **Concordant phenotypes** | | | **Investigator 1** | | **Investigator 2** | | **Investigator 3** | |
| --- | --- | --- | --- | --- | --- | --- | --- | --- |
|  |  |  | **Spearman’s Rank Correlation** | **Cohen’s Kappa** | **Spearman’s Rank Correlation** | **Cohen’s Kappa** | **Spearman’s Rank Correlation** | **Cohen’s Kappa** |
| **Photo-ageing** | | | | | | | | |
| Photo-ageing as measured by the Griffiths scale | Photo-ageing as measured by the Larnier scale | Value | 0.527 | 0.374 | 0.674 | 0.445 | 0.627 | 0.376 |
|  |  | p-Value | **2.41E-78** | **1.41E-64** | **4.53E-144** | **2.47E-111** | **2.70E-119** | **2.75E-78** |
| Photo-ageing as measured by the Griffiths scale | Photo-ageing as measured by the Korean scale (wrinkles constituent) | Value | 0.585 | 0.428 | 0.635 | 0.491 | 0.799 | 0.769 |
|  |  | p-Value | **2.79E-100** | **1.34E-88** | **2.53E-123** | **2.38E-122** | **6.31E-241** | **2.53E-230** |
| Photo-ageing as measured by the Griffiths scale | Photo-ageing as measured by the Korean scale (dyspigmentation constituent) | Value | 0.148 | 0.086 | 0.274 | 0.086 | 0.319 | 0.157 |
|  |  | p-Value | **1.00E-06** | **2.00E-05** | **5.06E-20** | **1.70E-05** | **6.31E-27** | **1.47E-16** |
| Photo-ageing as measured by the Larnier scale | Photo-ageing as measured by the Korean scale (wrinkles constituent) | Value | 0.544 | 0.321 | 0.665 | 0.408 | 0.64 | 0.419 |
|  |  | p-Value | **3.92E-84** | **2.66E-52** | **4.28E-139** | **8.45E-95** | **1.35E-125** | **2.54E-88** |
| Photo-ageing as measured by the Larnier scale | Photo-ageing as measured by the Korean scale (dyspigmentation constituent) | Value | 0.33 | 0.083 | 0.295 | 0.093 | 0.343 | 0.14 |
|  |  | p-Value | **7.95E-29** | **3.20E-05** | **4.22E-23** | **9.00E-06** | **4.03E-31** | **7.86E-11** |
| Photo-ageing as measured by the Korean scale (wrinkles constituent) | Photo-ageing as measured by the Korean scale (dyspigmentation constituent) | Value | 0.168 | 0.027 | 0.263 | 0.167 | 0.319 | 0.165 |
|  |  | p-Value | **2.66E-08** | **1.26E-01** | **1.47E-18** | **3.05E-12** | **6.01E-27** | **1.52E-17** |
